# Full-length NF-κB repressing factor contains an XRN2 binding domain

**DOI:** 10.1042/BCJ20190733

**Published:** 2020-02-27

**Authors:** Jana Alexandrova, David Piñeiro, Rebekah Jukes-Jones, Ryan Mordue, Mark Stoneley, Anne E. Willis

**Affiliations:** MRC Toxicology Unit, University of Cambridge, Lancaster Rd, Leicester LE1 9HN, U.K.

**Keywords:** protein synthesis, ribosome biogenesis, RNA-binding proteins, XRN2, XTBD

## Abstract

NF-κB repressing factor (NKRF) was recently identified as an RNA binding protein that together with its associated proteins, the 5′–3′ exonuclease XRN2 and the helicase DHX15, is required to process the precursor ribosomal RNA. XRN2 is a multi-functional ribonuclease that is also involved in processing mRNAs, tRNAs and lncRNAs. The activity and stability of XRN2 are controlled by its binding partners, PAXT-1, CDKN2AIP and CDKN2AIPNL. In each case, these proteins interact with XRN2 via an XRN2 binding domain (XTBD), the structure and mode of action of which is highly conserved. Rather surprisingly, although NKRF interacts directly with XRN2, it was not predicted to contain such a domain, and NKRF's interaction with XRN2 was therefore unexplained. We have identified an alternative upstream AUG start codon within the transcript that encodes NKRF and demonstrate that the full-length form of NKRF contains an XTBD that is conserved across species. Our data suggest that NKRF is tethered in the nucleolus by binding directly to rRNA and that the XTBD in the N-terminal extension of NKRF is essential for the retention of XRN2 in this sub-organelle. Thus, we propose NKRF regulates the early steps of pre-rRNA processing during ribosome biogenesis by controlling the spatial distribution of XRN2 and our data provide further support for the XTBD as an XRN2 interacting motif.

## Introduction

Ribosomes are complex macromolecular machines that translate the genetic code into functional polypeptides. They are comprised of four ribosomal RNAs (rRNA) and ∼80 proteins, and their biogenesis is one of the most energy consuming cellular processes [1]. Ribosome biogenesis is initiated by RNA polymerase I (RNAPI), which transcribes a single 47S precursor ribosomal RNA (pre-rRNA). The pre-rRNA is subsequently processed extensively through the insertion of 2′-O-methylations and pseudouridines guided by snoRNAs and base-specific modifications [2,3]. The full-length transcript is then subject to endonucleolytic cleavage and processing events to excise and degrade the 5′ and 3′ external transcribed spacers and internal transcribed spacers 1 and 2, to produce the mature 28S, 18S and 5.8S rRNAs [2]. The concerted actions of numerous RNA helicases, AAA-ATPases and GTPases are required for the remodelling of the rRNAs to form the correct tertiary structures present in the mature ribosome, to facilitate the recruitment or release of ribosome biogenesis factors and for the assembly of ribosomal proteins on to the rRNA [4–6].

Recently, using a combination of RNA affinity purification and proteomics approaches, a subset of RNA binding proteins (RBPs) were identified that showed reduced interaction with RNA following inhibition of RNAPI [7]. This group of 211 nuclear RBPs, termed the RNAPI RNA interactome, was found to be highly enriched for nucleolar proteins and proteins associated with ribosome biogenesis [7]. Within this group of rRNA biogenesis factors was NF-κΒ repressing factor (NKRF), which has been shown to participate in both the cleavage of precursor rRNA at the A′ site and clearance of the excised spacer fragments [8,9]. NKRF functions within a complex that additionally contains the 5′–3′ exonuclease, XRN2, and the DEAH-box RNA helicase, DHX15 [9].

XRN2 is multi-functional in terms of its RNA processing ability. In addition to its role in pre-rRNA processing, XRN2 is also involved in the degradation of aberrant pre-mRNA products, tRNAs and non-coding RNA processing [10,11]. Importantly, these alternative activities of XRN2 are dependent upon its interacting partners, which regulate its location and stability. Thus the interaction of XRN2 with PAXT-1 from *C. elegans*, which can be substituted by the functionally related human CDKN2AIPNL (C2AIL), regulates XRN2's stability [12,13], whereas its interaction with CDKN2AIP retains XRN2 in the nucleoplasm [14]. In these three examples, XRN2 interacts with its partner proteins through a specific XRN2-binding domain (XTBD), whose structure has been determined. It was proposed that the XTBD constitutes a general XRN2 interacting motif whose mode of action with XRN2 is conserved across the animal phylogeny [13]. Rather surprisingly, therefore, although NKRF was shown to bind to XRN2, this protein appeared to lack the majority of the XTBD in humans [8,9]. Consequently, Memet et al. suggested that the nature of binding of XRN2 with NKRF was likely to differ from that with CDKN2AIP or C2AIL [9].

Herein, we have identified an upstream in-frame AUG codon within the transcript that encodes human NKRF, which gives rise to an extended protein product of 784 amino acids (88 kDa) consistent with the molecular mass of this protein as determined by western blotting. Most importantly, we show that the full-length version of NKRF mRNA contains an XTBD that is conserved across species. We have evaluated the contribution of this N-terminal extension of NKRF to XRN2 localisation and we show that this binding domain is required for the retention of XRN2 in the nucleolus. These data suggest that NKRF controls the spatial distribution of XRN2 and provide further support for the role of the XTBD as a general XRN2 interacting motif.

## Experimental

### Cell culture, constructs and stable cell lines

U-2 OS and HeLa cells were grown at 37°C with 5% CO_2_. U-2 OS were cultured in Dulbecco's modified Eagle's medium (DMEM) supplemented with 10% FBS (Sigma–Aldrich). HeLa and U-2 OS cells were treated with 10 nM actinomycin D (Sigma–Aldrich) or 2.5 µM CX-5461 (Selleckchem).

The U-2 OS Flp-In T-REx cell line was generated using the manufacturer's recommended protocol (Thermo Fisher Scientific). The coding regions of the alternative versions of NKRF were inserted in frame with the N-terminal 3xFLAG tag in pCDNA5/FRT/TO (Thermo Fisher Scientific). Constructs were then transfected into the U-2 OS Flp-In T-REx cell line and stable clones were selected with 100 µg/ml hygromycin B (Invitrogen) and 2.5 µg/ml blasticidin (Corning).

### Western analysis and immunoprecipitation

For western analysis, proteins were separated on a 4–12% SDS–PAGE gel (Thermo Fisher Scientific), transferred on to PVDF membrane (Bio-Rad) and detected with the indicated antibodies (NKRF, GeneTex GTX105380 and Bethyl Laboratories A304-016A), XRN2 (Bethyl Laboratories A301-101A and A301-103A), nucleolin (GeneTex, GTX13541).

To prepare nuclei, U-2 OS cells were incubated in CLB buffer (10 mM HEPES pH 7.5, 10 mM NaCl, 3 mM MgCl_2_, 0.35 M sucrose, 0.5% NP-40) with protease inhibitors (cOmplete, Roche) for 5 min on ice and then centrifuged at 1300 ***g*** for 5 min at 4°C. The nuclear pellet was washed twice with CLB and incubated for 30 min in SNEB buffer (20 mM HEPES pH 7.5, 150 mM NaCl, 1% NP40, 0.5% deoxycholate, 0.1% SDS) with protease inhibitors, with or without 0.2 U/ml Benzonase (Novagen), as indicated. The extract was then cleared by centrifugation.

For immunoprecipitation, U-2 OS cells were lysed in CLB buffer (10 mM HEPES pH 7.5, 10 mM NaCl, 3 mM MgCl_2_, 0.35 M sucrose, 0.5% NP-40) with proteinase inhibitors (cOmplete, Roche), for 10 min on ice, then centrifuged at 1300 ***g*** for 5 min at 4°C. The nuclear pellet was then resuspended in lysis buffer L200 (50 mM Tris–HCl pH 7.4, 1% Triton X-100, 150 mM NaCl) sonicated, and where indicated was treated with 100U of RNase I (Ambion) or 8U of TurboDNase (Ambion). Immunoprecipitation was performed with 3 µg of anti-NKRF (Bethyl Laboratories, A304-016A) or anti-FLAG M2 (Sigma–Aldrich, F1804) for 2 h at 4°C. Proteins were eluted in 2× SDS–PAGE loading buffer. NKRF isolation for mass spectrometry identification was carried out in mildly denaturing conditions. U-2 OS cells were directly lysed in RIPA buffer (50 mM Tris–HCl pH 8, 150 mM NaCl, 0.5% sodium deoxycholate, 0.1% SDS, 1% NP-40) and subjected to 2 h incubation with anti-NKRF antibody (Bethyl Laboratories, A304-016A).

### RNA interactome capture (RIC)

RIC [15] was performed on nuclear extracts of U-2 OS. Briefly, cells were treated with 10 nM actinomycin D, 2.5 µM CX-5461 or DMSO. Cells were washed with ice-cold PBS, and cross-linked with UVC (150 mJ/cm^2^) irradiation. Nuclei were isolated as described above and were resuspended in oligo(dT) binding buffer (20 mM Tris pH 7.4, 500 mM LiCl, 0.5% LiDS, 1 mM EDTA, 5 mM DTT). Nuclear lysate was incubated with magnetic oligo(dT) beads (NEB) for one hour at room temperature. Subsequently, the beads were washed and the RNA was eluted from the beads (4). The eluted RNA was supplemented with 2 mM MgCl_2_, 125U Benzonase (Sigma–Aldrich) and 300U RNase I (Ambion) to digest the RNA.

### Mass spectrometry

LC–MS/MS was used to identify NKRF. Briefly, NKRF was immunoprecipiated and the samples were separated by SDS PAGE and serial gel slices digested *in situ* with trypsin. Extracted tryptic peptides were analysed using data-independent acquisition (DIA) on a nanoAcquity UPLC system coupled to a Waters Synapt G2-S HDMS mass spectrometer. The PLGS ‘TOP 3’ method was used for absolute quantification of proteins.

### Immunolocalization

U-2 OS or HeLa cells were seeded on to glass coverslips and after the indicated treatments were fixed in 4% paraformaldehyde and permeabilized with 0.2% Triton X-100. Primary antibody incubations were performed at 4°C overnight with the indicated antibodies: NKRF (GeneTex, GTX105380), XRN2 (Bethyl Laboratories, A301-103A), fibrillarin (Abcam, ab5821 and ab4566), anti-FLAG M2 (Sigma–Aldrich, F1804). Secondary fluorescent antibodies were incubated for 1 h at room temperature (Alexa Fluor 488 anti-Rabbit, Thermo Fisher Scientific, A11008; Alexa Fluor 568 anti-Rabbit, Thermo Fisher Scientific, A11011; Alexa Fluor 488 anti-Mouse, Thermo Fisher Scientific, A11001; Alexa Fluor 568 anti-Mouse, Thermo Fisher Scientific, A11004). Finally, the nuclei were labelled with Hoechst 33342 (Thermo Fisher Scientific). Images were acquired on Zeiss 710 confocal microscope and analysed with ImageJ.

## Results

### Identification of an extended mRNA encoding full-length NKRF

**Figure 1.**
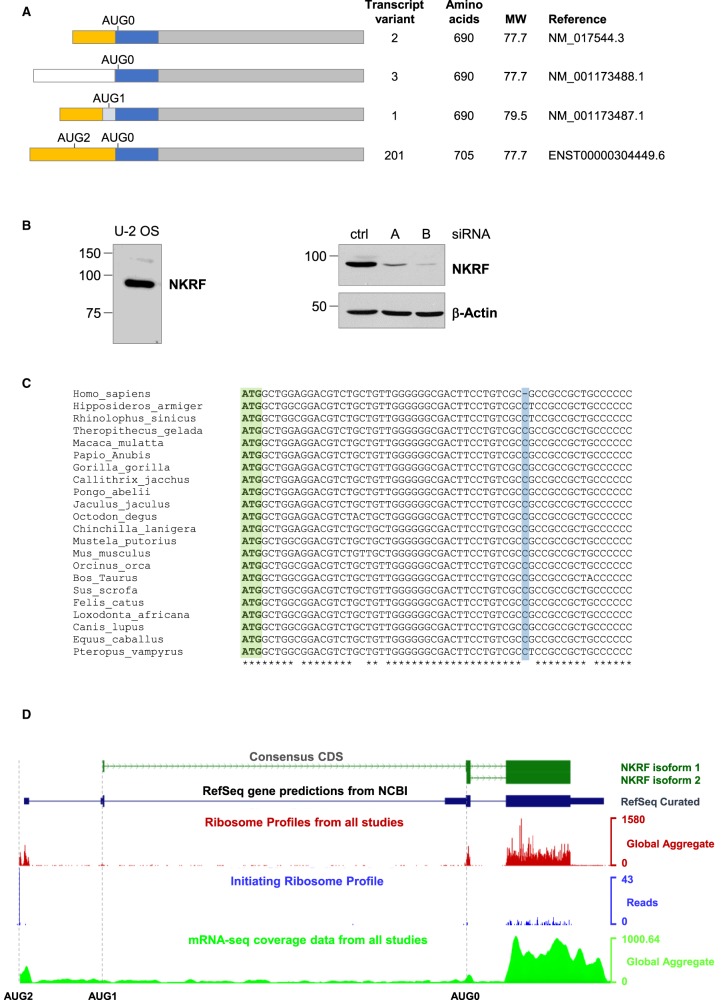
The full-length NKRF transcript encodes a protein of 784 amino acids. (**A**) Schematic representation of the NKRF mRNA variants identified in the sequence databases. The three main exons are represented in orange, blue and grey. AUG0 is initiation codon for the short NKRF protein; AUG1 is initiation codon for the version derived from mRNA variant 1 that contains an additional 15 amino acids; AUG2 is the initiation codon for the full-length NKRF identified herein. (**B**) Western analysis of U-2 OS cell extracts reveals that NKRF protein migrates at 88 kDa and not at the predicted molecular mass of 77 kDa. RNAi-mediated depletion was used to reduce the cellular abundance of NKRF using two NKRF siRNAs (A,B) and a control siRNA (ctrl). Western analysis confirmed that the 88 kDa protein is depleted by both NKRF siRNAs demonstrating that the antibody specifically recognises NKRF protein. (**C**) The sequences of NKRF from different mammals were compared. Green denotes the upstream initiation codon AUG2, and blue the cytosine nucleotide missing from the human reference genomic sequence. (**D**) Ribosome profiling experiments were analysed and visualised using the Genome Wide Information on Protein Synthesis tool (GWIPS-viz) [31] to determine the position of translating ribosomes on the NKRF mRNA. The global aggregate of ribosome footprint reads from 43 ribosome profiling studies was plotted against the NKRF gene sequence to generate a ribosome density map of elongating ribosomes on the NKRF gene (red profile). As expected, ribosome density can be detected in the previously annotated coding exons 2 and 3. However, ribosome density also extends into and upstream of the previously described exon 1, which was assigned as non-coding, as far as the new initiation codon AUG2 (red profile). Enrichment of ribosome density can be seen at AUG2, but not at AUG1 or AUG0, after treatment of cells with homoharrintonine (blue plot) [18]. RNA-seq reads (green profile) indicate that the NKRF mRNA extends further upstream than the RefSeq mRNA database entry (NM_0017544.3). The grey dotted lines highlight the position of AUG2, AUG1 and AUG0.

NKRF, which was recently shown to have a role in ribosome biogenesis [8,9], was originally identified as a 43.8 kDa protein that repressed the transcriptional activity of NF-κB and constitutively silenced the IFN-β promoter [16]. However, sequencing of additional cDNA clones and analysis of genomic sequences from murine and human tissues revealed a sequencing error in the first published NKRF cDNA sequence, which led to premature termination [16]. A new corrected sequence was proposed, which encoded for a 302 amino acid extension at the C-terminal end of the open reading frame, giving rise to a protein of 690 amino acids (Figure 1A) with a predicted molecular mass of 77 kDa [17]. The data suggested that NKRF protein was encoded by two mRNAs, differing in their 5′UTRs (Figure 1A: variants 2 and 3), and in addition, an alternative splice variant, which could produce a protein of 705 amino acids, was also proposed (Figure 1A: variant 1). However, none of these predicted protein sizes are consistent with the 88 kDa protein corresponding to NKRF that we observed by western analysis (Figure 1B). To confirm the specificity of the NKRF antibody used in western analysis, we attempted to deplete NKRF in U-2 OS cells using two different siRNAs directed against NKRF mRNA. Both siRNAs caused a significant decrease in the 88 kDa protein, confirming that the antibody detects NKRF (Figure 1B). In addition, a similar reduction in NKRF expression in the nucleolus was observed after siRNA depletion (Supplementary Figure S1A). Although it is possible that the migration of NKRF protein is affected by post-translational modifications, in the first instance, we carried out extensive bioinformatic analysis to understand the discrepancy between the predicted and observed protein size. We identified an extended mRNA encoding NKRF (Figure 1A: transcript variant 201), which was found by automatic annotation in Ensembl as ENST00000304449.6. The 5′ untranslated region (5'UTR) of this mRNA is 560 nucleotides longer than that of the previously described variant 2 transcript, and importantly, this longer 5′UTR contains an upstream AUG codon (Figure 1A: AUG2). The automatic translation of this sequence still gave a reference sequence protein of 690 amino acids (77 kDa). However, the dbSNP short genetic variations database revealed that the human NKRF genomic reference sequence contains an error. Thus 99.95% of the 20 387 human genomes sequenced contained an additional cytosine nucleotide in exon 1 of the NKRF gene when compared with the reference sequence (Figure 1C and Supplementary Figure S1B). Inclusion of the additional cytosine shifts AUG2 in the longer NKRF mRNA into the same reading frame as the previously identified coding region, extending the open reading frame to encode a 784 amino acid protein with a predicted molecular mass of 88 kDa. Importantly, the amino acid sequence of this N-terminal extension is highly conserved between mammalian species (Supplementary Figure S1C) with all corresponding genomic sequences containing an additional cytosine when compared with the human reference sequence.

Analysis of RNA sequence datasets from different tissues and cell lines confirmed the presence of the extended NKRF mRNA (Figure 1D: green profile). Ribosome profiling, a technique that accurately defines the position of translating ribosomes on the mRNA, identified ribosome density within NKRF exon 1 and translation initiation from AUG2. Thus, a global analysis of 43 ribosome profiling experiments revealed significant ribosome footprints in the NKRF mRNA that extend upstream of AUG0 and AUG1 and as far as AUG2 (Figure 1D, red profile). Indeed, in these experiments ribosome density can be found upstream of the previously assigned 5′ end of transcript variant 2 covering the extended coding sequence that we identified. Furthermore, analysis of initiating ribosome profiling data, in which ribosomes are arrested at the initiation codon using homoharringtonine [18], revealed a significant enrichment of ribosome density at AUG2, supporting the recognition of this initiation codon *in vivo* (Figure 1D, blue profile). To further validate these data morpholino oligonucleotides were designed that would sterically block translation complexes initiating from either AUG2 or from AUG0. However, neither of the morpholino oligonucleotides were able to inhibit the synthesis of NKRF protein (data not shown), suggesting that such an approach was incompatible with the cell system used. Nevertheless, when taken together the data strongly suggest that translation initiation occurs at AUG2 in the extended NKRF mRNA, resulting in the production of full-length NKRF.

### Full-length NKRF contains an XTBD

**Figure 2.**
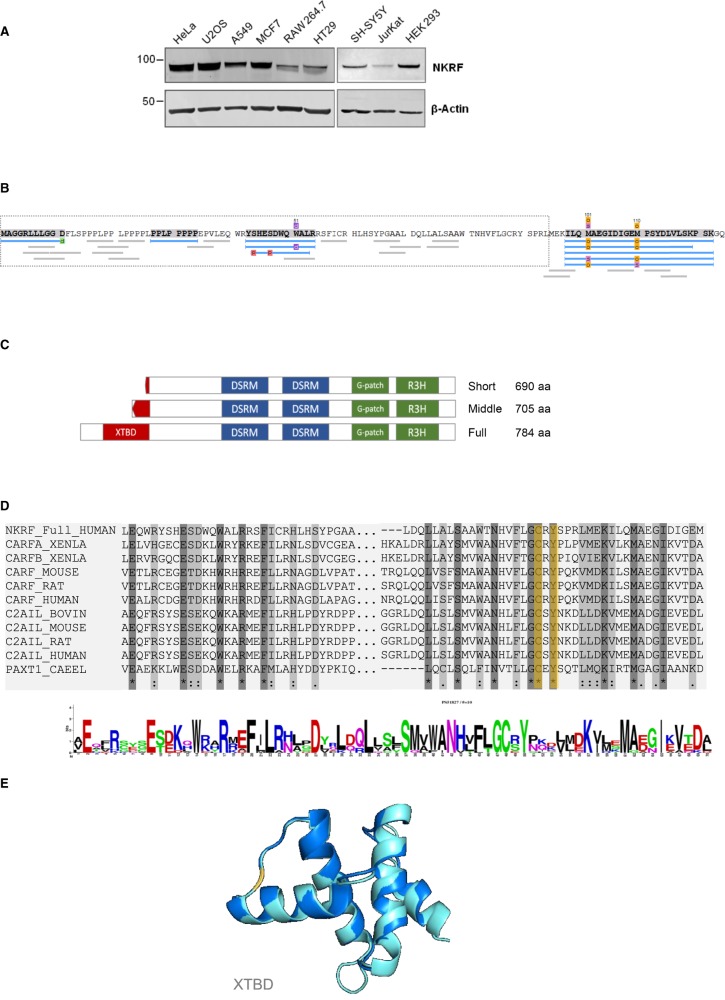
Full-length NKRF contains an XRN2 binding domain (XTBD). (**A**) Cell extracts from HeLa, U-2 OS, A549, MCF7, RAW264.7, HT29, SH-SY5Y, Jurkat and HEK293 cells were separated by SDS PAGE and subjected to western analysis using an antibody against NKRF. Actin was used as a loading control. (**B**) NKRF was immunoprecipitated and subjected to mass spectrometry which identified full-length NKRF (NKRF_full) containing an intact XTBD domain. The sequence of the N-terminus of the extended NKRF is depicted with the sequence that is unique to NKRF_full in the grey dotted box. The three sequences that are unique to NKRF_full that were identified by mass spectrometry are highlighted in grey. Peptides that were identified in these experiments are represented as blue lines. (**C**) A schematic presentation of NKRF proteins is shown representing (i) the short isoform of 690 amino acids, (ii) the middle isoform of 705 amino acids, and (iii) the full-length protein of 784 amino acids. All isoforms of NKRF contain three RNA binding domains: G-Patch, RH3, and double strand RNA binding domain. Only full-length NKRF contains the intact XRN2 binding domain (red). (**D**) The NKRF XRN2 binding domain (XTBD) contains functionally important conserved residues. Sequence comparison of XTBD from CDKN2AIP (from mouse, rat and human), C2AIL (CDKN2AIPNL) (from mouse, rat, bovine and human), PAXT-1 (*C. elegans*) and human full-length NKRF shows a high degree of conservation and particularly residues Cys54 and Tyr56 (orange), which are essential for the interaction with XRN2. (**E**) A structural model of the NKRF XTBD domain was generated using RaptorX [32] and this structure was superimposed on the crystal structure of PAXT-1 using PyMol. The residues Cys54 and Tyr56, which are essential for the interaction with XRN2, are shown in orange.

To determine the extent of full-length NKRF expression across cell types, extracts from cells representative of lung cancer (A549), breast cancer (MCF7), colorectal cancer (HT29), osteosarcoma (U-2 OS), cervical cancer (HeLa), neuroblastoma (SH-SY5Y), T-lymphocytes (Jurkat), macrophages (RAW264.7) and embryonic kidney (HEK293) were subjected to western analysis. In each case, only an NKRF protein of 88 kDa was detected (Figure 2A). To confirm that NKRF protein contains the extended N-terminus, the endogenous protein was immunoprecipitated under mildly denaturing conditions from U-2 OS and HeLa cells and the isolated proteins were subjected to mass spectrometry (Figure 2B and Supplementary Table S1). Three peptides were identified with a high confidence score that are unique to the N-terminally extended NKRF (MAGGRLLLGGD, PPLPPPPP, and YSHESDWQWALRR) (Figure 2B and Supplementary Table S1). These data confirm both the bioinformatic analysis, that NKRF is longer than previously defined, and importantly, that NKRF contains the entire 75 amino acids of the XTBD. We term the full-length protein NKRF_full (Figure 2C).

The published sequence of the previously described short version of human NKRF contained only 11 amino acids of the XTBD, and based on previous elegant structural studies, this region would be insufficient for its binding to XRN2 [13]. Importantly, sequence comparison between the full-length XTBD of NKRF with PAXT-1, CDKN2AIP and CDKN2AIPNL shows the conservation of key residues (Figure 2D); for example, Tyr56 and Cys54, which are required for the interaction with XRN2 (Figure 2D, shown in yellow). A 3D model structure for NKRF XTBD was generated using RaptorX, which was then superimposed onto the PAXT-1 XTBD crystal structure (Figure 2E). The degree of structural conservation is remarkable, again supporting the hypothesis that NKRF contains a functional XTBD. Moreover, a comparison of NKRF protein sequences from different mammalian species shows almost 100% conservation of this domain (Supplementary Figure S1C).

### NKRF binds directly to XRN2 and this interaction is required for XRN2 localisation

**Figure 3.**
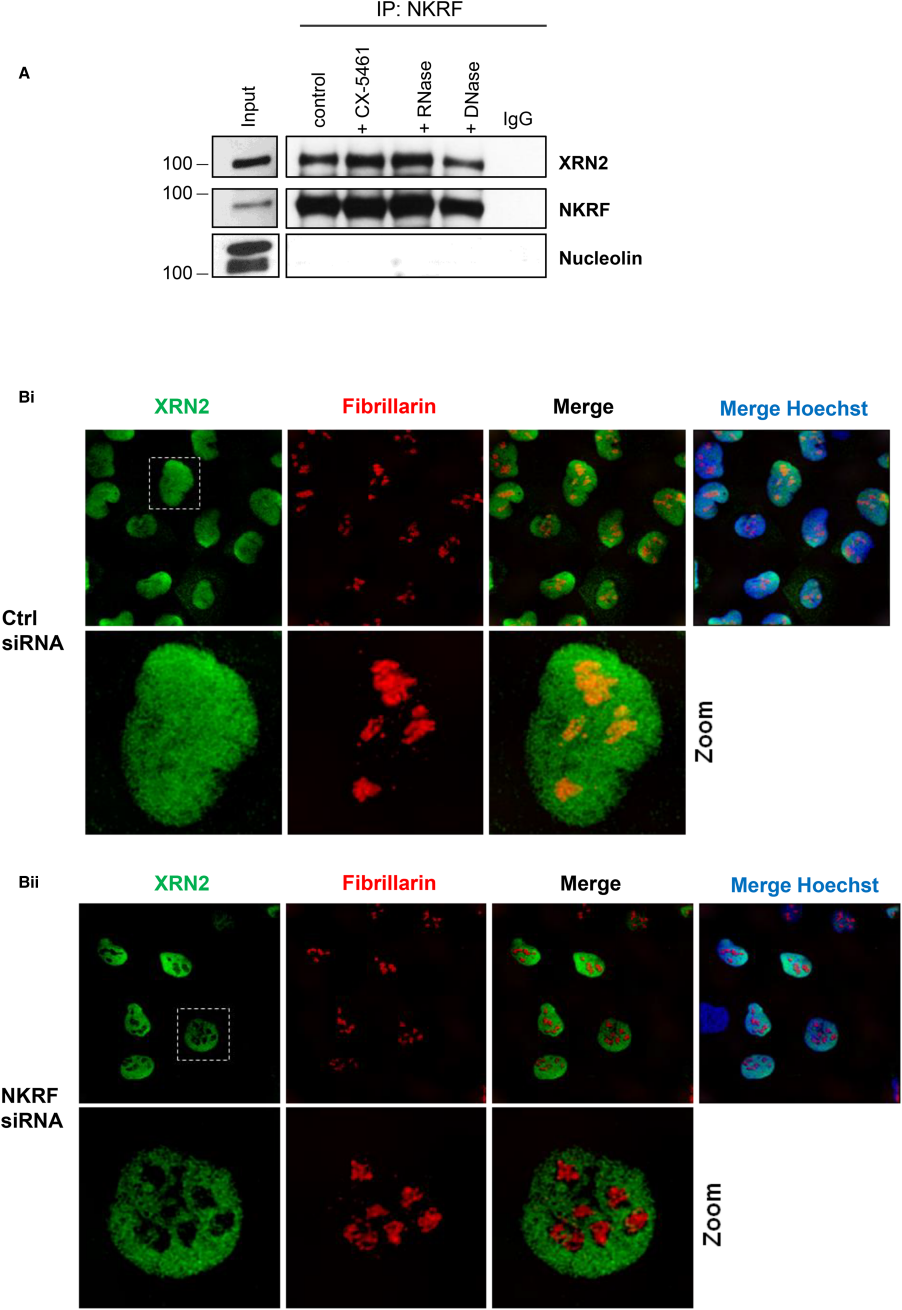
The interaction between NKRF and XRN2 is independent of rRNA. (**A**) U-2 OS cells were treated with the specific RNAPI inhibitor CX-5461 (CX) and cell extracts were generated from untreated and treated cells. Untreated cell lysates were incubated with either RNase or DNase for 2 h at 4°C. NKRF was then immunoprecipitated using an anti-NKRF antibody or the control IgG. The corresponding samples were separated by SDS–PAGE and immunoblotted with antibodies directed against NKRF and XRN2. Nucleolin was used as negative control. (**B**) U-2 OS cells were transfected with control siRNA (i) or siRNA targeting NKRF (ii) and after 72 h cells were fixed and permeabilised. XRN2 cellular distribution was determined by immunolocalisation. XRN2 is visualised in green; Hoechst (blue) and fibrillarin (red) were used as nuclear and nucleolar markers, respectively.

The presence of an XTBD in the N-terminus of NKRF suggests that NKRF and XRN2 can interact directly through protein–protein interactions [7]. To confirm that this interaction occurs *in vivo*, endogenous NKRF was immunoprecipitated from U-2 OS cells. Western analysis of the NKRF immuno-isolates confirmed that XRN2 could be efficiently co-purified with NKRF (Figure 3A). Since both NKRF and XRN2 are pre-rRNA binding proteins [7] we also performed NKRF immunoprecipitations from cells treated with the RNAPI inhibitor CX-5461 or from lysates treated with nucleases. There was no effect on the recovery of XRN2 in the NKRF immuno-isolates after nuclease digestion or RNAPI inhibition confirming that this interaction is not dependent on nucleic acids (Figure 3A), in agreement with previous studies [9]. To determine whether the presence of XRN2 in the nucleolus was dependent on NKRF, the level of NKRF protein was reduced by siRNA depletion and immunofluorescence and confocal microscopy were used to identify changes in XRN2 subcellular localisation (Figure 3B). In control cells, XRN2 was detected in the nucleoplasm, and in addition, a proportion of XRN2 co-localised with fibrillarin in the nucleolus (Figure 3Bi). However, depletion of NKRF protein resulted in the loss of XRN2 from the nucleolus, as shown by the lack of XRN2 co-localisation with the nucleolar marker fibrillarin (Figure 3Bi,ii). Therefore, the accumulation of XRN2 in the nucleolus depends on NKRF.

### Inhibition of RNA polymerase I (RNAPI) releases both NKRF and XRN2 from the nucleolus

**Figure 4.**
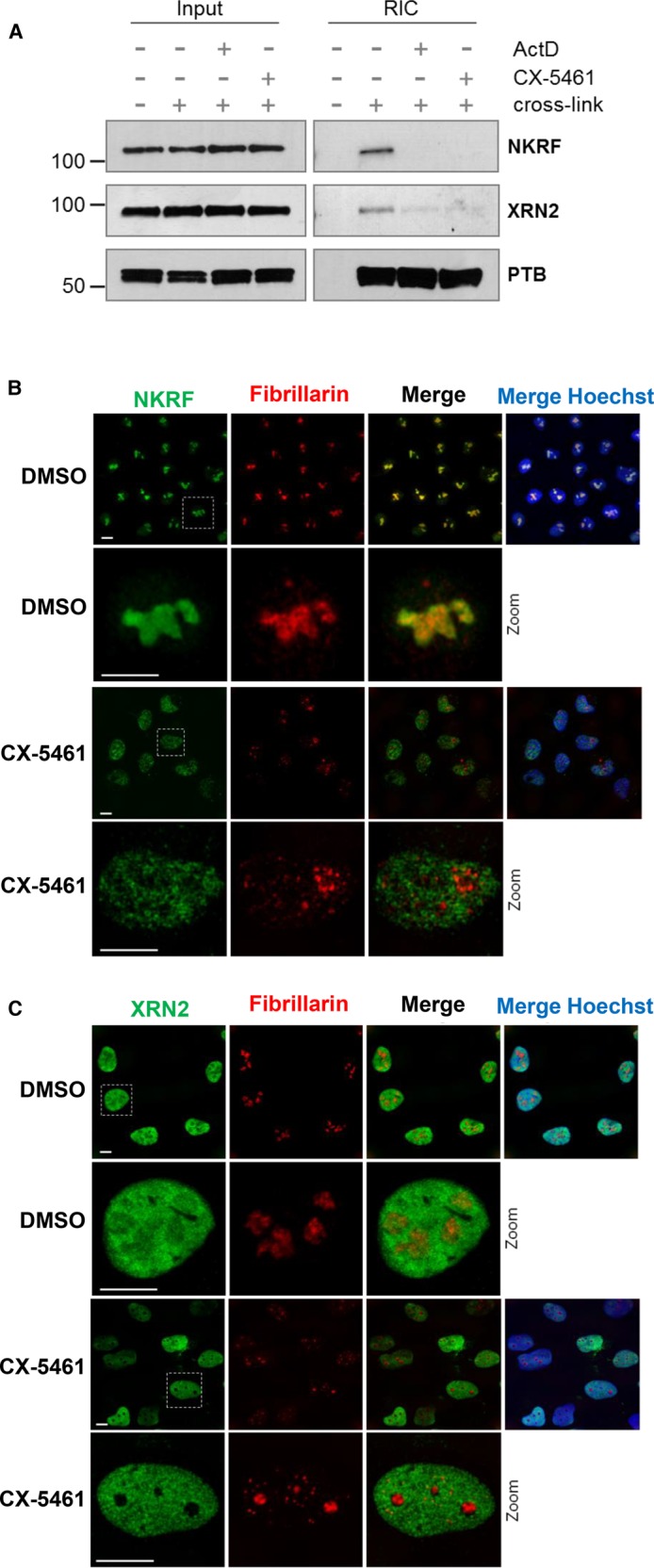
Co-localization of NKRF and XRN2 is dependent on RNAPI activity. (**A**) RNA interactome capture was performed on U-2 OS cells that were either untreated or treated with a low dose of actinomycin or 2.5 µM CX-5641 to inhibit RNAPI. The samples were immunoblotted and probed with antibodies against XRN2 and NKRF. Polypyrimidine tract binding protein (PTB) was used as a control. (**B**) U-2 OS cells were either untreated or exposed to 2.5 µM CX-5461 and immunolocalization was performed. The subcellular distribution of either NKRF (green) or fibrillarin (red) was determined using specific antibodies. Hoechst (blue) and fibrillarin (red) were used as nuclear and nucleolar marker, respectively. (**C**) U-2 OS cells either untreated or exposed to 2.5 µM CX-5461 and immunolocalization was performed. The subcellular distribution of either XRN2 (green) or fibrillarin (red) was determined using specific antibodies. Hoechst (blue) and fibrillarin (red) were used as nuclear and nucleolar marker, respectively.

A recent proteomic analysis cataloguing RNA–protein interactions that are disrupted after the inhibition of RNAPI identified NKRF as a candidate RNAPI-dependent RBP, suggesting that NKRF interacts with RNAPI transcripts. To confirm these findings, we used RNA-interactome capture (RIC) to determine the effect of RNAPI inhibition on NKRF and XRN2 RNA binding (Figure 4A). Both NKRF and XRN2 were recovered in the RIC assay after cross-linking, but not in the absence of cross-linking, confirming that these proteins can interact with RNA. Moreover, inhibition of RNAPI activity using either actinomycin D or CX-5461 reduced the interaction of both NKRF and XRN2 with RNA, suggesting that these proteins bind to rRNA. It was then important to investigate whether the presence of NKRF and XRN2 in the nucleolus was dependent on their interaction with rRNA. Therefore, RNAPI activity was inhibited by treating U-2 OS cells with CX-5461 and the subcellular distribution of NKRF and XRN2 was determined by immunofluorescence and confocal microscopy (Figure 4B,C). Upon RNAPI inhibition, NKRF protein redistributes from the nucleoli into the nucleoplasm and, under the same conditions, XRN2 is also lost from the nucleolus. Thus, both the nucleolar localisation and the rRNA binding activity of NKRF and XRN2 depend on continued transcription of pre-rRNA. These data, in conjunction with the observation that the nucleolar localisation of XRN2 requires NKRF, suggest a model in which NKRF binds to rRNA to tether XRN2 in the nucleolus.

### NKRF XTBD is required for efficient binding of NKRF to XRN2 and XRN2 nucleolar localisation

**Figure 5.**
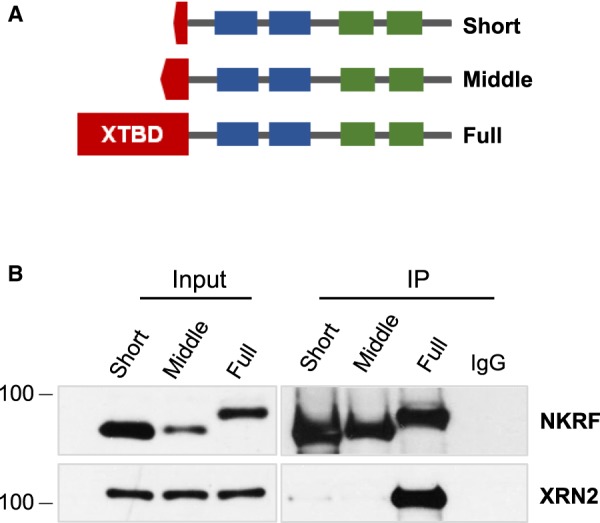
Full-length NKRF containing the XTBD interacts with XRN2. (**A**) Schematic to show the three variants of NKRF that were expressed in U-2 OS cells. The expressed proteins are tagged at the N-terminus with a 3xFLAG tag. (**B**) U-2 OS cells were transiently transfected with the constructs expressing short, middle or full-length NKRF containing an N-terminal expressed FLAG tag. Cell extracts were generated and proteins were immunoprecipitated using anti-FLAG antibody. IgG was used as negative control. Isolated proteins were separated by SDS–PAGE and subjected to western analysis.

To determine the importance of the XTBD in the direct interaction of NKRF with XRN2, a series of plasmid constructs was generated that express the 784 (full), 705 (middle) and 690 (short) amino acid versions of the protein with an N-terminal FLAG tag (Figure 5A). U-2 OS cells were transiently transfected with the plasmid constructs, cell extracts were immunoprecipitated using anti-flag antibodies, and samples were separated by SDS–PAGE and immunoblotted for NKRF and XRN2 (Figure 5B). Strikingly, only full-length NKRF containing the entire XTBD was able to bind XRN2 efficiently, showing that this domain is essential for the interaction between NKRF and XRN2 (Figure 5B).

**Figure 6.**
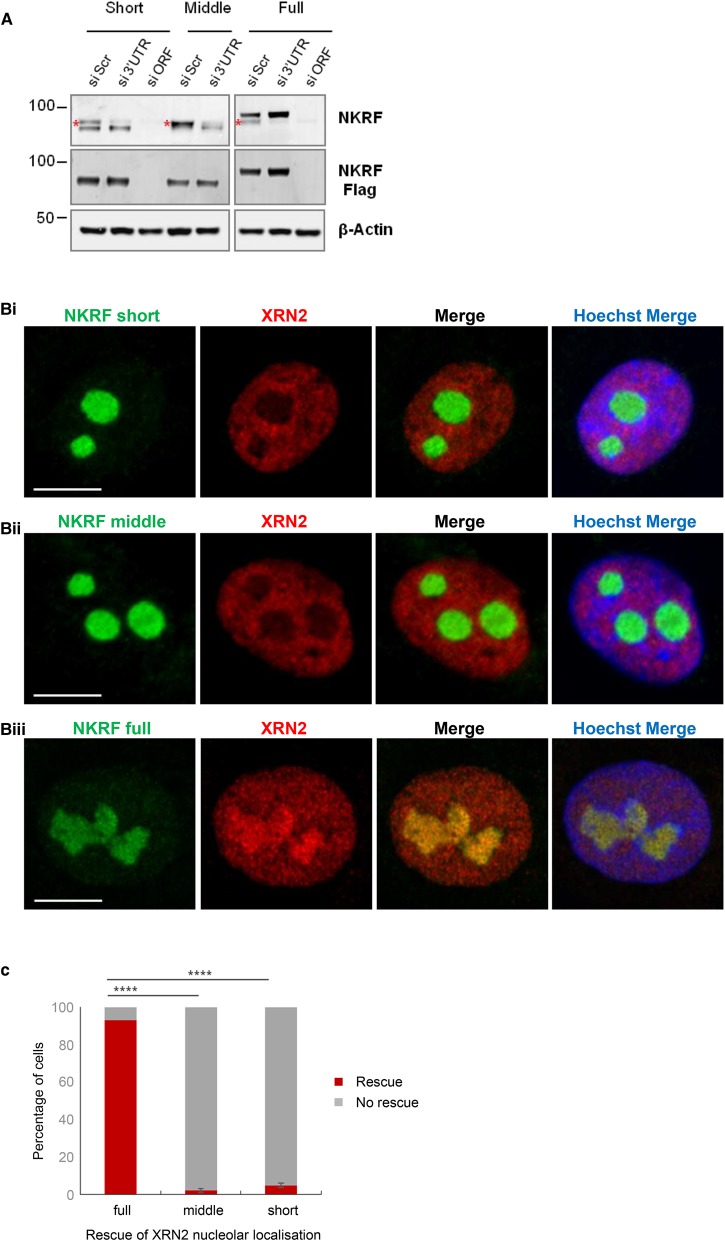
Full-length NKRF containing the XTBD restores the nucleolar localization of XRN2. (**A**) NKRF short, middle and full-length U-2 OS stable cell lines were transfected with an siRNA to the NKRF open reading frame (siORF), which would target both the endogenous and expression versions of the proteins, or the NKRF 3′UTR (si3′UTR), which would only target endogenous NKRF. After 72 h, the expression of the NKRF isoforms was induced with tetracycline for a further 24 h. Cell extracts were generated and immunoblotted with antibodies against either FLAG or NKRF. Endogenous NKRF is marked (red asterisk). (**B**) NKRF short (i), middle (ii) and full-length (iii) U-2 OS stable cell lines were transfected with siRNAs targeting the 3′UTR of endogenous NKRF. After 72 h, expression of the FLAG-tagged versions of NKRF was induced for 24 h with tetracycline. Cells were fixed and permeabilised and the subcellular distribution of XRN2 (red) was determined using immunolocalization. NKRF (green) was visualised using anti-FLAG antibodies. Nuclei were stained with Hoechst (blue). (**C**) The data in (**B**) were quantified by counting ∼100 cells per condition to assess the XRN2 distribution in the nucleoli after expression of the different version of NKRF. The percentage of cells where XRN2 was present in the nucleoli was calculated (*n* = 3, error bars represent SD, and the significance was calculated using Fisher's exact test).

As discussed previously, NKRF is predominately a nucleolar protein, whereas XRN2 in present in both the nucleolus and the nucleoplasm, and it has been proposed that NKRF plays a key role in anchoring XRN2 in the nucleoli and controlling its bioavailability. To investigate the role of the XTBD in targeting XRN2 to the nucleolus, stable cell lines were generated which expressed FLAG-tagged short, middle and full-length versions of NKRF. Importantly, each of the tagged proteins localised to the nucleolus and the full-length tagged protein was lost from the nucleolus after inhibition of RNAPI, confirming that the tagged proteins behave in a similar manner to the endogenous protein in these cell lines (Supplementary Figure S2). Endogenous NKRF was depleted in these stable cell lines using siRNAs (Figure 6A) and the expression of the FLAG-tagged versions of the proteins was induced with tetracycline (Figure 6B and Supplementary Figure S3). The cellular distribution of XRN2 was assessed by immunolocalization to determine whether it was possible to rescue the loss of XRN2 from the nucleolus by expressing each of the NKRF protein isoforms (Figure 6Bi–iii,C and Supplementary Figure S3). Despite the fact that both NKRF short and middle are targeted to the nucleolus (Supplementary Figure S2Ai–ii), expression of neither of these NKRF isoforms rescued the nucleolar localisation of XRN2 (Figure 6Bi–ii,C and Supplementary Figure S3). In contrast, the full-length version of NKRF containing the entire XTBD domain was able to restore XRN2 to the nucleoli (Figure 6C and Supplementary Figure S3C). These data demonstrate that the XTBD in NKRF is essential for targeting XRN2 to the nucleolus, thereby allowing XRN2 to participate in pre-ribosomal RNA processing.

## Discussion

Spatial control of multi-functional RBPs is required for a large number of key cellular processes including ribosome biogenesis, stress signalling and cell cycle progression [19–23]. For example, NKRF functions as a RNAPII transcription termination factor in the nucleoplasm [24–28] and in nucleolus where it has a role in ribosome biogenesis [7]. Previous studies suggested that NKRF is tethered in the nucleolus by binding to rRNA since its presence in this sub-compartment is dependent upon the activity of RNAPI [7]. The activity of RNAPI is inhibited by cell stress [21] and NKRF relocates into the nucleoplasm following exposure of cells to wide range of stresses e.g. heat exposure [8]. Other work suggested that NKRF interacts with XRN2, targeting the exonuclease to the nucleolus and thereby enabling the correct processing of pre-ribosomal RNA [7]. However, the precise details of XRN2 nucleolar localisation by NKRF were not fully understood since NKRF appeared to lack the essential XRN2 interaction surface (XTBD) [8,9,13,14]. However, we have now identified an N-terminal extension to the NKRF protein that contains a highly conserved XTBD (Figures 1, 2) [13,14]. This extended protein was not identified previously due to an error in both the cDNA and genomic sequence that resulted in an upstream AUG codon (Figure 1A: AUG2) being out of frame with the coding sequence. Importantly, both ribosome profiling data and mass spectrometry analysis confirmed that this extended polypeptide is produced in human cells (Figure 1D, 2B and Supplementary Table S1). While this manuscript was in preparation, an updated mRNA sequence appeared in the NCBI database that corrected this sequencing error (NM_017544.5), further supporting the existence of the extended NKRF protein. However, the sequencing error persists in the Ensembl database (ENST00000304449.6) and in the RefSeq genomic database.

In agreement with others, we find that NKRF interacts with XRN2 (Figure 3A) [8,9,29]. However, we also show that efficient binding of NKRF to XRN2 absolutely depends on the integrity of the XTBD (Figure 5A). Furthermore, we find that NKRF targets XRN2 to the nucleolus (Figure 3B) [8], but in addition, our data demonstrate that nucleolar accumulation of XRN2 requires an intact XTBD (Figure 6B,C). Therefore, it seems likely that the function of the XTBD is to target the XRN2 5′–3′ exonuclease to the nucleolus.

It has been proposed that NKRF interacts with the pre-rRNA and contributes to ribosomal RNA processing [9]. RNA interactome capture experiments confirmed that NKRF RNA binding depends almost entirely on RNAPI activity, suggesting that NKRF does indeed interact with pre-rRNA (Figure 4A). Furthermore, we also observed a concomitant reduction in XRN2 RNA binding after RNAPI inhibition (Figure 4A). This significant reduction in RNA binding correlated with the loss of both of these proteins from the nucleolar sub-compartment (Figure 4B,C), and coupled with the observation that XRN2 nucleolar localisation requires NKRF, suggests a simple model for the interplay between NKRF and XRN2. NKRF is targeted to the nucleolus through its interaction with pre-rRNA, and in turn, the XTBD in NKRF recruits XRN2 to the nucleolus and to the pre-rRNA allowing the XRN2 exonuclease to interact with and process the pre-rRNA.

In vertebrates, there are only two other proteins that contain an XTBD, CDKN2AIP and C2AIL [14]. Interestingly one of these proteins, CDKN2AIP, is also an RBP that appears to oppose the function of NKRF with respect to XRN2 subcellular targeting. Thus, increased expression of CDKN2AIP correlates with the accumulation of XRN2 in the nucleoplasm and cellular depletion of CDKN2AIP enhances targeting of XRN2 to the nucleolus [14]. These data suggest that NKRF and CDKN2AIP positively and negatively regulate, respectively, the early steps of pre-rRNA processing during ribosome biogenesis by the spatial control of XRN2 through their XTBDs. Therefore, alterations in the relative activities of NKRF and CDKN2AIP would control ribosome biogenesis and contribute to increased cell growth and in some circumstances could drive tumorigenesis. In this regard, in malignant melanoma it has been shown that altered expression of the long non-coding RNA (lncRNA) SAMMSON sequesters CDKN2AIP in an aberrant cytoplasmic RNA–protein complex, which promotes XRN2 localisation to the nucleoli, stimulating rRNA biogenesis, protein synthesis, and tumour cell growth [30].

## Open Access

Open access for this article was enabled by the participation of University of Cambridge in an all-inclusive *Read & Publish* pilot with Portland Press and the Biochemical Society under a transformative agreement with JISC.

## Abbreviations

C2AIL: CDKN2AIPNL
NKRF: NF-κB repressing factor
pre-rRNA: precursor ribosomal RNA
RIC: RNA interactome capture
RNAPI: RNA polymerase I
RBPs: RNA binding proteins
rRNA: ribosomal RNA
XTBD: XRN2 binding domain
