# Supplementary material for: Full-length NF-κB repressing factor contains an XRN2 binding domain
Source: Biochem J. 2020 Feb 27;477(4):773–86. doi: 10.1042/BCJ20190733 (PMC7054742; doi:10.1042/BCJ20190733)
Supplement: Supplementary Figures S1-S3 and Table S1 [file BCJ-477-773-s1.pdf]

Supplementary Figure Legends

Supplementary Figure 1: RNAi-mediated depletion of endogenous NKRF

A) U-2 OS cells were transfected with 10 nM of siRNA targeting the open reading frame (siNKRF) or a control siRNA. Cells were fixed, permeabilised and used for immunolocalization with specific antibodies. Hoechst (blue) and fibrillarin (red) were used as nuclear and nucleolar marker respectively. NKRF is visualized in green.

B) Single nucleotide polymorphism rs5903560 from NCBI Short Genetic Variations database shows that an additional cytosine nucleotide is present in 99.95 % of the tested genomic sequences (20663 genomes from the Genome Aggregation Database). Therefore, AUG2 is in-frame with the rest of the coding region.

C) Sequence comparison of NKRF N-terminal sequence from different mammals showing the conservation of the XTBD domain (yellow).

Supplementary Figure 2: NKRF expression in stable cell lines

A) Immunolocalization of short, middle and full-length version of NKRF using anti-FLAG antibodies. U-2 OS stable cell lines were induced with tetracycline to express the short (i), middle (ii), and full-length (iii) versions of the NKRF protein. Cells were fixed and permeablised and immunolocalization was performed. NKRF is visualized in green and XRN2 in red. Hoechst (blue) was used as a nuclear marker.

B) Expression of full-length NKRF was induced using tetracycline in a stable cell line and cells were treated with the RNAPI inhibitor CX-5461. Cells were fixed and permeablised and immunolocalization was performed. NKRF is visualized in green and fibrillarin in red. Hoechst (blue) was used as a nuclear marker. The data show that the FLAG-tagged full-length version of NKRF requires RNAPI activity to accumulate in the nucleolus.

Supplementary Figure 3. Full-length NKRF is required for localization for XRN2

U-2 OS stable cell lines expressing the short **(A)**, middle **(B)** and full **(C)** length versions of NKRF were transfected with siRNAs targeting the 3'UTR of endogenous NKRF. Cells were fixed and permeabilised and the presence of XRN2 (red) in the nucleoli and nucleoplasm was detected using specific antibodies. NKRF (green) was visualized using anti-FLAG antibodies. The nuclei are stained with Hoechst (blue).

Supplementary Table 1

Sequences of peptides identified by mass spectrometry are shown, relative to their position within full-length NKRF.

A

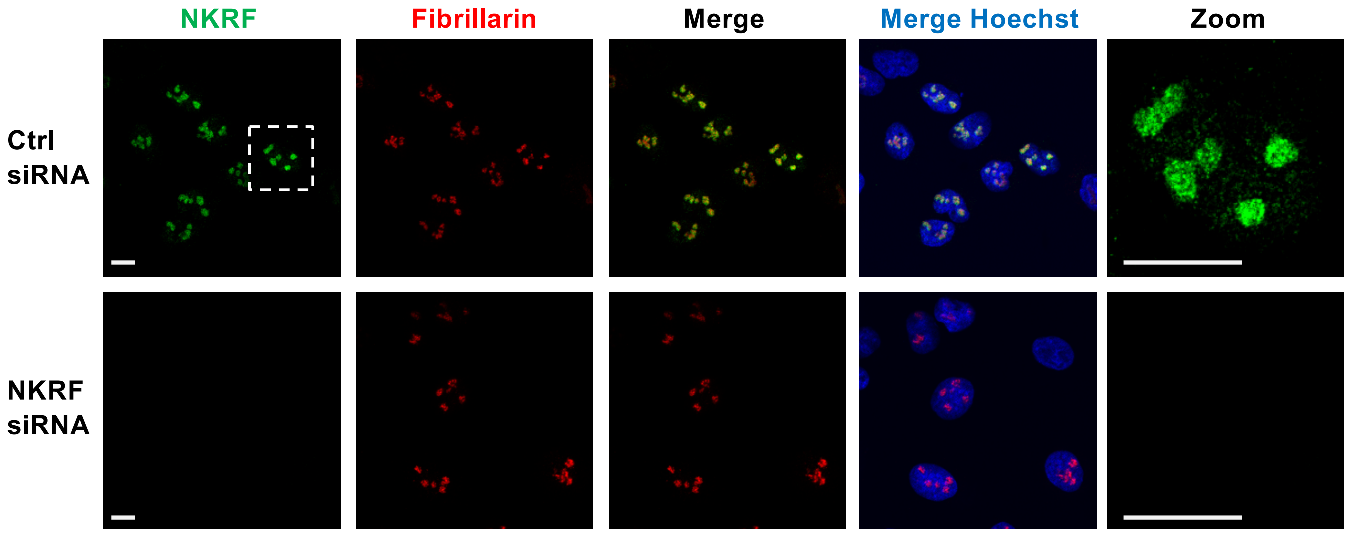

B

rs5903560 [Homo sapiens]

GGGGGGGCAGCGGCGGC[/G]GCGAC

Chromosome: X:119605952

Gene: NKRF

Allele frequency: **0.9995**

C

```
A0A2I3M155_PAPAN    MAGGRLLLGGDFLSPPPLPPLPPPPLPPLPPPPPEPVLEQWRYSHESDWQWALRRSFICRHLHSYPGAALDQLLALSAAWTNHVFLGCRYSPRLMEKILQMAEGIDIGEMPSYDLVLSKP
A0A0P6JLU8_HETGA    MAGGRLLLGGDFLSPPPLPPLPPPPLPPLPPPPPEPVLEQWRYSHESDWQWALRRSFICRHLHSYPGAALDQLLALSAAWTNHVFLGCRYSPRLMEKILQMAEGIDIGEMPSYDLMLSKT
A0A091CQ04_FUKDA    MAGGRLLLG--------------------PPPPPEPVLEQWRYSHESDWQWALRRSFICQHLHSYPGAALDQLLALSAAWTNHVFLGCRYSPRLMEKILQMAEGIDIGEMPSYDLMLSKP
S7P740_MYOBR        MAGGRLLLGGDFLSPPPLLPLPPP------PPPPDPVLEQWRYSHESDWQWALRRSFICRHLHSYPGAALDQLLALSAAWTNHVFLGCRYSPRLMEKILQMAEGIDIGEMPSYDLMLSKP
M3XR33_MUSPF        MAGGRLLLGGDFLSPPPLPPLPPPPLPPLPPPPPEPVLEQWRYSHESDWQWALRRSFICRHLHSYPGAALDQLLALSAAWTNHVFLGCRYSPRLMEKILQMAEGIDIGEMPSYDLMLSKP
A0A2Y9KK35_ENHLU    MAGGRLLLGGDFLSPPPLPPLPPPPLPPLPPPPPEPVLEQWRYSHESDWQWALRRSFICRHLHSYPGAALDQLLALSAAWTNHVFLGCRYSPRLMEKILQMAEGIDIGEMPSYDLMLSKP
L5L608_PTEAL        MAGGRLLLGGDFLSPPPLPPLPPPPPPPLPPPPPEPVLEQWRYSHESDWQWALRRSFICRHLHSYPGAALDQLLALSAAWTNHVFLGCRYSPRLMEKILQMAEGIDIGEMPSYDLMLSKP
F1RUA0_PIG          MAGGRLLLGGDFLSPPPLPPLPPPPLPPLPPPPPEPVLEQWRYSHESDWQWALRRSFICRHLHSYPGAALDQLLALSAAWTNHVFLGCRYSPRLMEKILQMAEGIDIGEMPSYDLMLSKP
A0A2Y9MCC7_DELLE    MAGGRLLLGGDFLSPPPLPPLPPPPLPPLPPPPPEPVLEQWRYSHESDWQWALRRSFICRHLHSYPGAALDQLLALSAAWTNHVFLGCRYSPRLMEKILQMAEGIDIGEMPSYDLMLSKP
A0A2U3V4G5_TURTR    MAGGRLLLGGDFLSPPPLPPLPPPPLPPLPPPPPEPVLEQWRYSHESDWQWALRRSFICRHLHSYPGAALDQLLALSAAWTNHVFLGCRYSPRLMEKILQMAEGIDIGEMPSYDLMLSKP
A0A2U3WAB6_ODORO    MAGGRLLLGGDFLSPPPLPPLPPPPLPPLPPPPPEPVLEQWRYSHESDWQWALRRSFICRHLHSYPGAALDQLLALSAAWTNHVFLGCRYSPRLMEKILQMAEGIDIGEMPSYDLMLSKP
M3WA55_FELCA        MAGGRLLLGGDFLSPPPLPPLPPPPLPPLPPPPPEPVLEQWRYSHESDWQWALRRSFICRHLHSYPGAALDQLLALSAAWTNHVFLGCRYSPRLMEKILQMAEGIDIGEMPSYDLMLSKP
E2RI67_CANLF        MAGGRLLLGGDFLSPPPLPPLPPPPLPPLPPPPPEPVLEQWRYSHESDWQWALRRSFICRHLHSYPGAALDQLLALSAAWTNHVFLGCRYSPRLMEKILQMAEGIDIGEMPSYDLMLSKP
F7EGU6_CALJA        MAGGRLLLGGDFLSPPPLPPLPPPPLPPLPPPPPEPVLEQWRYSHESDWQWALRRSFICRHLHSYPGAALDQLLALSAAWTNHVFLGCRYSPRLMEKILQMAEGIDIGEMPSYDLMLPKS
A0A0D9S0H2_CHLSB    MAGGRLLLGGDFLSPPPLPPLPPPPLPPLPPPPPEPVLEQWRYSHESDWQWALRRSFICRHLHSYPGAALDQLLALSAAWTNHVFLGCRYSPRLMEKILQMAEGIDIGEMPSYDLVLSKP
A0A2K6B606_MACNE    MAGGRLLLGGDFLSPPPLPPLPPPPLPPLPPPPPEPVLEQWRYSHESDWQWALRRSFICRHLHSYPGAALDQLLALSAAWTNHVFLGCRYSPRLMEKILQMAEGIDIGEMPSYDLVLSKP
H2PWL7_PONAB        MAGGRLLLGGDFLSPPPLPPLPPPPLPPLPPPPPEPVLEQWRYSHESDWQWALRRSFICRHLHSYPGAALDQLLALSAAWTNHVFLGCRYSPRLMEKILQMAEGIDIGEMPSYDLVLSKP
                    *********                     ****:***********************:*******************************************************:* *
```

Figure S1

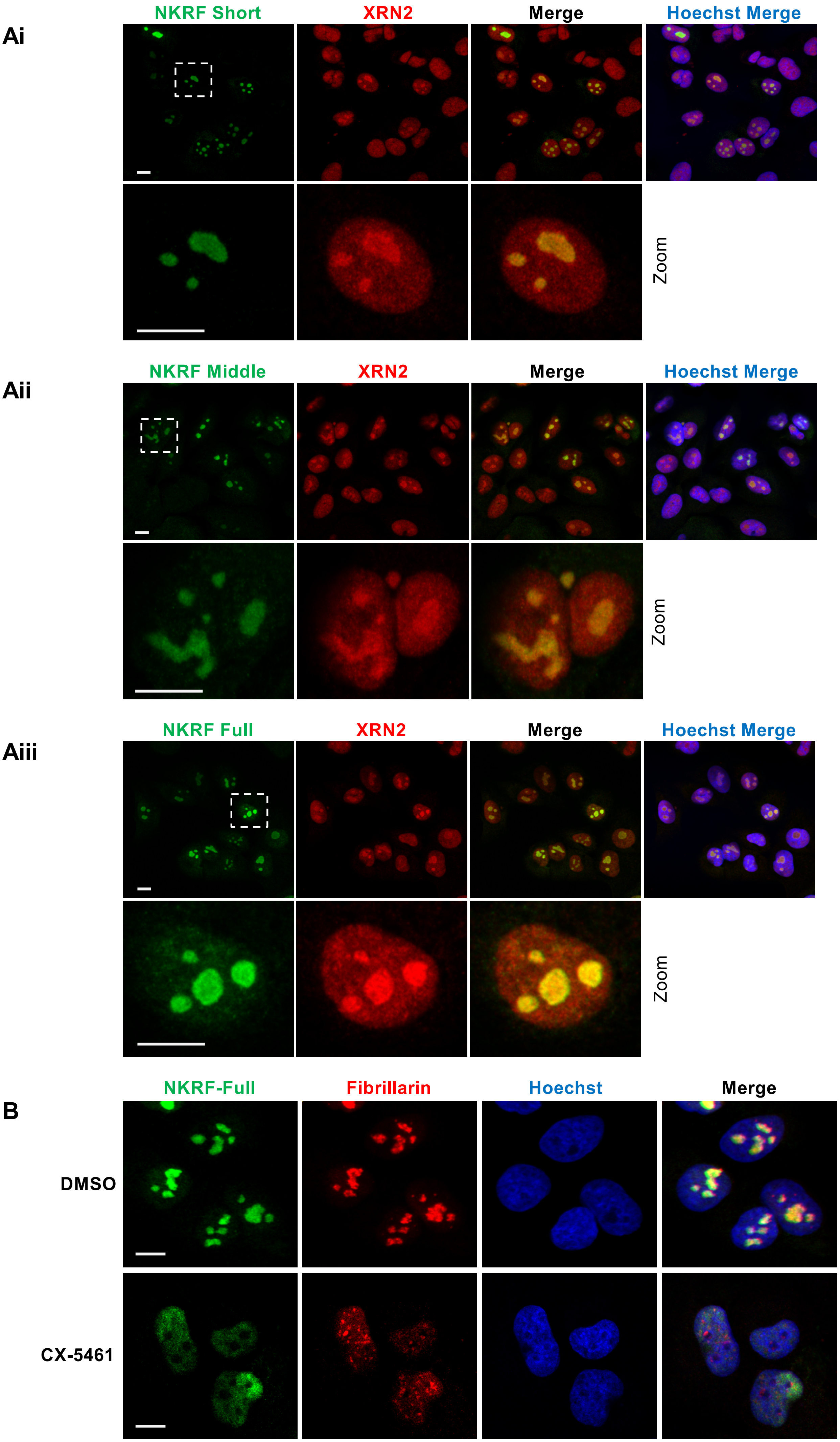
Ai
NKRF Short
XRN2
Merge
Hoechst Merge
Zoom
Aii
NKRF Middle
XRN2
Merge
Hoechst Merge
Zoom
Aiii
NKRF Full
XRN2
Merge
Hoechst Merge
Zoom
B
NKRF-Full
Fibrillarin
Hoechst
Merge
DMSO
CX-5461

Figure S2

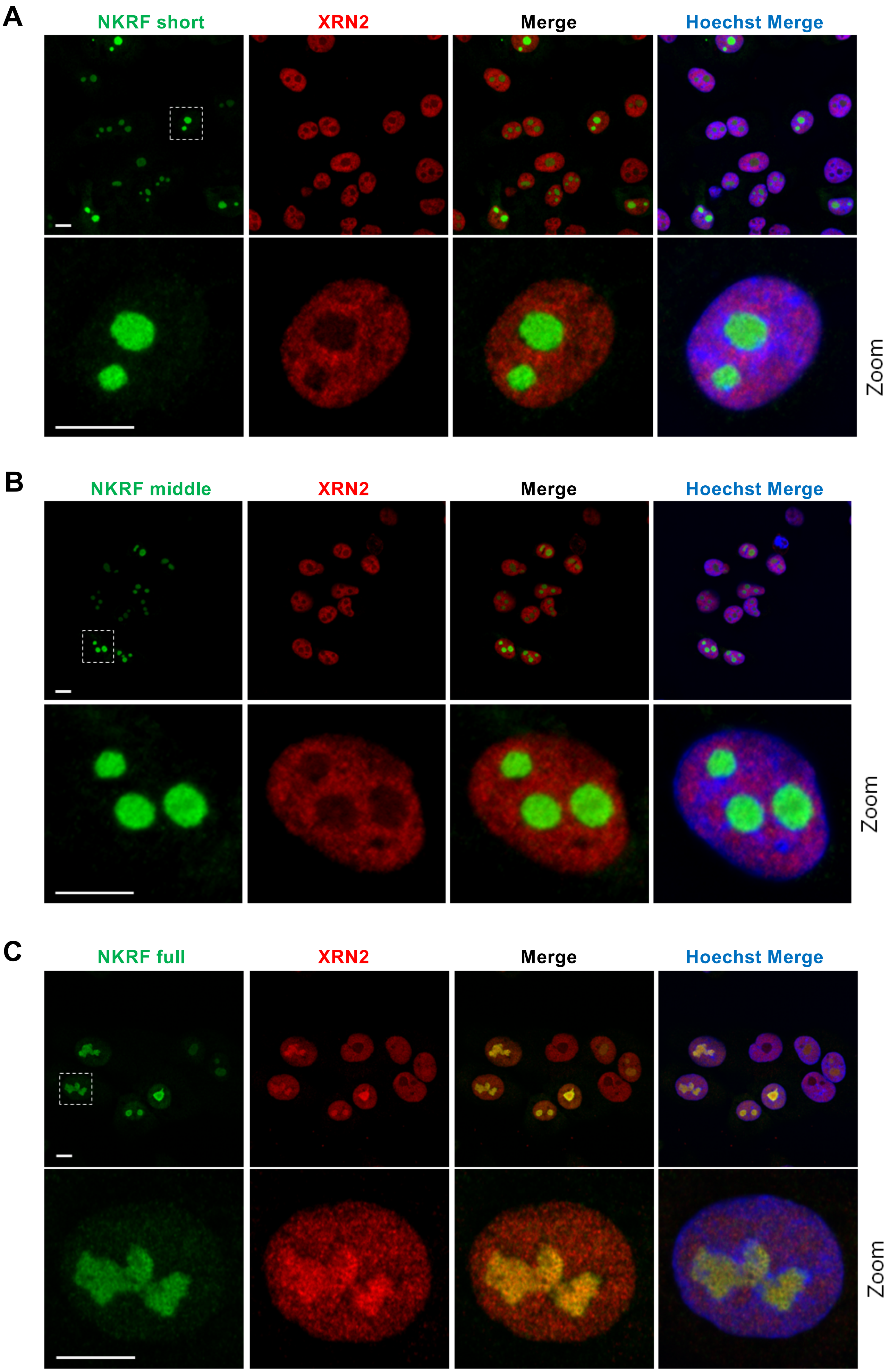
A
NKRF short
XRN2
Merge
Hoechst Merge
Zoom
B
NKRF middle
XRN2
Merge
Hoechst Merge
Zoom
C
NKRF full
XRN2
Merge
Hoechst Merge
Zoom

Figure S3

Supplementary Table 1

Protein ID	Peptide	-10lgP	Start	End
NKRF_HUMAN	MAGGRLLLGGD(-18.01).F	15.42	1	11
NKRF_HUMAN	L.PPLPPPPP.E	18.46	27	34
NKRF_HUMAN	R.YSHESDWQWALR.R	60.25	43	54
NKRF_HUMAN	R.YSHESDWQW(+31.99)ALR.R	25.07	43	54
NKRF_HUMAN	Y.S(+79.97)HES(+79.97)DWQWAL.R	17.31	44	53
NKRF_HUMAN	K.ILQM(+15.99)AEGIDIGEM(+15.99)PSYDLVLSKPSK.G	98.66	98	122
NKRF_HUMAN	K.ILQM(+15.99)AEGIDIGEM(+15.99)PSYDLVLSK.P	55.81	98	119
NKRF_HUMAN	K.ILQM(+31.99)AEGIDIGEM(+15.99)PSYDLVLSKPSK.G	40.62	98	122
NKRF_HUMAN	K.ILQM(+15.99)AEGIDIGEM(+31.99)PSYDLVLSKPSK.G	39.63	98	122
NKRF_HUMAN	K.ILQMAEGIDIGEMPSYDLVLSKPSK.G	15.55	98	122
NKRF_HUMAN	K.RHLSTC(+47.98)DGQNPPKK.Q	43.73	126	139
NKRF_HUMAN	K.RHLSTC(+45.99)DGQ(+.98)NPPKK.Q	29.45	126	139
NKRF_HUMAN	K.RHLSTC(+43.04)DGQNPPKK.Q	19	126	139
NKRF_HUMAN	K.RHLS(+79.97)TCDGQNPPK.K	16.2	126	138
NKRF_HUMAN	R.HLSTCDGQNPPKK.Q	79.56	127	139
NKRF_HUMAN	R.HLSTC(+47.98)DGQNPPK.K	59.96	127	138
NKRF_HUMAN	R.HLSTC(-33.99)DGQNPPKK.Q	57.11	127	139
NKRF_HUMAN	R.HLSTC(+47.98)DGQNPPKK.Q	56.16	127	139
NKRF_HUMAN	R.HLSTC(+47.94)DGQNPPKK.Q	52.6	127	139
NKRF_HUMAN	R.HLSTC(+47.94)DGQNPPK.K	49.95	127	138
NKRF_HUMAN	R.HLSTC(+45.99)DGQNPPKK.Q	44.15	127	139
NKRF_HUMAN	R.HLST(+87.05)CDGQNPPKK.Q	41.2	127	139
NKRF_HUMAN	R.HLST(-15.99)CDGQNPPKK.Q	36.32	127	139
NKRF_HUMAN	R.HLST(+87.05)CDGQNPPK.K	35.61	127	138
NKRF_HUMAN	R.HLSTC(+45.99)DGQ(+.98)NPPKK.Q	28.52	127	139
NKRF_HUMAN	R.HLSTCDGQNP(+31.99)PK.K	24.29	127	138
NKRF_HUMAN	R.HLS(-20.03)TCDGQNPPKK.Q	24.01	127	139
NKRF_HUMAN	R.HLSTC(-20.03)DGQNPPKK.Q	21.84	127	139
NKRF_HUMAN	R.HLST(+79.97)CD(+43.99)GQNPPK.K	21.82	127	138
NKRF_HUMAN	R.HLSTC(+45.99)DGQ(+.98)NPPK.K	20.63	127	138
NKRF_HUMAN	R.HLSTC(+45.99)DGQN(+.98)PPKK.Q	20.38	127	139
NKRF_HUMAN	R.HLSTC(+31.99)DGQNPPKK.Q	19.53	127	139
NKRF_HUMAN	R.HLSTC(+105.06)DGQN(+.98)PPK.K	17.55	127	138

NKRF_HUMAN	R.HLSTCDGQNPPKK(-2.02).Q	16.22	127	139
NKRF_HUMAN	R.HLST(-2.02)CDGQNPPKK.Q	15.87	127	139
NKRF_HUMAN	S.T(+79.97)CDGQNPPK.K	19.41	130	138
NKRF_HUMAN	K.KQAGS(+79.97)KFHARPR.F	15.48	139	150
NKRF_HUMAN	K.QAGS(+79.97)KFHARPR.F	16.08	140	150
NKRF_HUMAN	H.AR(+80.99)PRFEPVHFVASSSKDER.Q	22.61	147	165
NKRF_HUMAN	R.FEPVHFVASSSK.D	103.15	151	162
NKRF_HUMAN	R.FEPVHFVASSSKDER.Q	93.76	151	165
NKRF_HUMAN	R.FEPVHFVASSSKDERQEDPYGPQTK.E	44.63	151	175
NKRF_HUMAN	R.FEPVHF(+31.99)VASSSKDER.Q	38.97	151	165
NKRF_HUMAN	R.FEP(+31.99)VHFVASSSKDER.Q	26.71	151	165
NKRF_HUMAN	R.FEPVHFVASSSKDERQED.P	23.79	151	168
NKRF_HUMAN	R.FEPVHF(+31.99)VASSSK.D	20.99	151	162
NKRF_HUMAN	R.FEPVH(+14.02)FVASSSKDER.Q	19.08	151	165
NKRF_HUMAN	R.FEPVH(+14.02)FVASSSK.D	16.49	151	162
NKRF_HUMAN	R.FEPVHFVASS(+79.97)S(+79.97)K.D	16.46	151	162
NKRF_HUMAN	R.FEPVHFVASSS(+79.97)K.D	16.45	151	162
NKRF_HUMAN	F.EPVHFVASSSKDER.Q	37.67	152	165
NKRF_HUMAN	E.PVHFVASSSK.D	56.79	153	162
NKRF_HUMAN	E.PVHFVASSSKDER.Q	55.78	153	165
NKRF_HUMAN	K.DERQEDPYGPQTK.E	87.08	163	175
NKRF_HUMAN	K.DERQ(+.98)EDPYGPQTK.E	52.66	163	175
NKRF_HUMAN	K.DERQEDPY(+15.99)GPQTK.E	29.07	163	175
NKRF_HUMAN	K.DERQED(+17.03)PYGPQTK.E	24.45	163	175
NKRF_HUMAN	K.DERQED(+14.02)PYGPQTK.E	21.7	163	175
NKRF_HUMAN	K.DERQEDPYGPQTK(-.98).E	21.49	163	175
NKRF_HUMAN	R.QEDPYGPQTK.E	82.59	166	175
NKRF_HUMAN	R.Q(-17.03)EDPYGPQTK.E	65.24	166	175
NKRF_HUMAN	R.QED(-18.01)PYGPQTK.E	48.27	166	175
NKRF_HUMAN	R.Q(+42.01)EDPYGPQTK.E	46.62	166	175
NKRF_HUMAN	R.Q(+43.01)EDPYGPQTK.E	30.51	166	175
NKRF_HUMAN	R.QEDPY(+15.99)GPQTK.E	27.27	166	175
NKRF_HUMAN	R.QEDP(+13.98)YGPQTK.E	25.02	166	175
NKRF_HUMAN	R.QEDPYGPQ(+.98)TK.E	23.12	166	175

NKRF_HUMAN	R.QE(+21.98)DPYGPQTK.E	21.69	166	175
NKRF_HUMAN	R.QED(+21.98)PYGPQTK.E	19.2	166	175
NKRF_HUMAN	R.QEDPYGPQTK(+14.02).E	17.83	166	175
NKRF_HUMAN	R.Q(+42.01)EDPYGPQTK(+14.02).E	16.64	166	175
NKRF_HUMAN	R.Q(+.98)ED(+21.98)PYGPQTK.E	16.51	166	175
NKRF_HUMAN	Q.EDPYGPQTK.E	15.47	167	175
NKRF_HUMAN	D.PYGPQTK.E	22.01	169	175
NKRF_HUMAN	K.EVNEQTHFASM(+15.99)PR.D	106.39	176	188
NKRF_HUMAN	K.E(-18.01)VNEQTHFASM(+15.99)PR.D	80.77	176	188
NKRF_HUMAN	K.EVNEQTHFASM(+31.99)PR.D	67.05	176	188
NKRF_HUMAN	K.EVNEQ(+.98)THFASM(+15.99)PR.D	63.79	176	188
NKRF_HUMAN	K.EVN(+.98)EQTHFASM(+15.99)PR.D	56.22	176	188
NKRF_HUMAN	K.EVNEQTHF(+31.99)ASM(+15.99)PR.D	48.88	176	188
NKRF_HUMAN	K.EVNEQTHF.A	32.75	176	183
NKRF_HUMAN	K.EVNEQ(+.98)THFASM(+31.99)PR.D	29.32	176	188
NKRF_HUMAN	K.EVNEQTHFASM(+15.99)PR(+14.02).D	26.08	176	188
NKRF_HUMAN	K.EVNE(+21.98)QTHFASM(+15.99)PR.D	24.84	176	188
NKRF_HUMAN	K.E(+42.01)VNEQTHFASM(+15.99)PR.D	24.27	176	188
NKRF_HUMAN	K.EVNEQTH(+15.99)FASMPR(+14.02).D	19.63	176	188
NKRF_HUMAN	K.EVNEQTHFASMP(+31.99)R(+14.02).D	19.5	176	188
NKRF_HUMAN	K.EVNEQTHFAS(+162.05)MP.R	18.43	176	187
NKRF_HUMAN	K.EVNEQT(+79.97)HFASMPR.D	16.69	176	188
NKRF_HUMAN	E.VNEQTHFASM(+15.99)PR.D	72.51	177	188
NKRF_HUMAN	V.NEQTHFASM(+15.99)PR.D	61.81	178	188
NKRF_HUMAN	N.EQTHFASM(+15.99)PR.D	28.52	179	188
NKRF_HUMAN	R.DIYQDYTQDSF.S	59.95	189	199
NKRF_HUMAN	Y.C(+25.00)DSSGFILTK.D	23.71	209	218
NKRF_HUMAN	K.DQPVTANM(+15.99)YFDSGNPAPSTTSQQANSQSTPEPSPSQTFPESVVAEK.Q	54.27	219	264
NKRF_HUMAN	K.DQPVTANM(+15.99)YFDSGNPAPSTTSQQAN.S	54.03	219	243
NKRF_HUMAN	N.SQSTPEPSPSQTFPESVVAEK.Q	56.77	244	264
NKRF_HUMAN	K.LTATIWK.N	43.45	271	277
NKRF_HUMAN	K.LTATIW(+15.99)K.N	36.26	271	277
NKRF_HUMAN	K.LTATIW(+3.99)K.N	31.12	271	277
NKRF_HUMAN	K.L(+42.01)TATIWK.N	22.5	271	277

NKRF_HUMAN	K.LTATIW(+43.99)K.N	21.21	271	277
NKRF_HUMAN	K.LTATIW(+31.99)K.N	19.37	271	277
NKRF_HUMAN	K.LTATIWK(+14.96).N	16.12	271	277
NKRF_HUMAN	K.NLSNPEM(+15.99)TSGSDKINYTYM(+15.99)LTR.C	93.33	278	299
NKRF_HUMAN	K.NLSNPEM(+15.99)TSGSDK.I	89.6	278	290
NKRF_HUMAN	K.NLSNPEM(+31.99)TSGSDK.I	51.16	278	290
NKRF_HUMAN	K.N(+42.01)LSNPEM(+15.99)TSGSDK.I	49.83	278	290
NKRF_HUMAN	K.NLSNPEM(+31.99)TSGSDKINYTYM(+15.99)LTR.C	46.37	278	299
NKRF_HUMAN	K.NLSNP(+31.99)EMTSGSDKINYTYM(+15.99)LTR.C	32.73	278	299
NKRF_HUMAN	K.NLSNPE(+43.99)MTSGSDK.I	30.23	278	290
NKRF_HUMAN	K.NLSNPE(+21.98)M(+15.99)TSGSDK.I	25.46	278	290
NKRF_HUMAN	K.NLSNP(+31.99)EM(+15.99)TSGSDK.I	22.68	278	290
NKRF_HUMAN	K.NLSNPEM(+15.99)T(+79.97)SGSDK.I	22.48	278	290
NKRF_HUMAN	K.N(+27.99)LSNPEM(+15.99)TSGSDK.I	20.24	278	290
NKRF_HUMAN	K.N(+42.01)LS(+79.97)NPEMTSGSDK.I	17.28	278	290
NKRF_HUMAN	K.NLSNPE(+37.96)MTSGSDK.I	16.4	278	290
NKRF_HUMAN	K.NLSNPE(+14.02)MTSGSDK.I	16.13	278	290
NKRF_HUMAN	S.NPEMTS(+79.97)GSDK.I	17.72	281	290
NKRF_HUMAN	K.INYTYM(+15.99)LTR.C	61.9	291	299
NKRF_HUMAN	K.I(+42.01)NYTYM(+15.99)LTR.C	45.54	291	299
NKRF_HUMAN	K.INYTYM(+31.99)LTR.C	41.26	291	299
NKRF_HUMAN	K.INYTY(+33.96)MLTR.C	23.74	291	299
NKRF_HUMAN	K.INYT(+79.97)YM(+15.99)LTR.C	15.99	291	299
NKRF_HUMAN	R.CIQACKT(+95.94)NPEYIYAPLK.E	40.79	300	316
NKRF_HUMAN	R.C(+68.06)IQACKTNPEYIYAPLK.E	19.66	300	316
NKRF_HUMAN	R.CIQAC(+44.03)KTN(+.98)PEYIYAPLK.E	16.33	300	316
NKRF_HUMAN	K.TNPEYIYAPLK.E	88.42	306	316
NKRF_HUMAN	K.T(-18.01)N(+.98)PEYIYAPLK.E	49.76	306	316
NKRF_HUMAN	K.TNPEYIYAPLKEIPPADIPK.N	46.64	306	325
NKRF_HUMAN	K.T(+42.01)NPEYIYAPLK.E	45.06	306	316
NKRF_HUMAN	K.TNPE(+28.03)YIYAPLK.E	31.23	306	316
NKRF_HUMAN	K.TN(+.98)PEYIYAPLKEIPPADIPK.N	18.54	306	325
NKRF_HUMAN	K.T(+27.99)N(+.98)PEYIYAPLK.E	17.73	306	316
NKRF_HUMAN	K.T(+27.99)NPEYIYAPLK.E	16.46	306	316

NKRF_HUMAN	K.T(+79.97)NPEYIYAPLK.E	15.99	306	316
NKRF_HUMAN	T.N(+127.06)PEYIYAPLK.E	37.68	307	316
NKRF_HUMAN	N.PEYIYAPLK.E	45.64	308	316
NKRF_HUMAN	K.EIPPADIPK.N	57.12	317	325
NKRF_HUMAN	K.E(-18.01)IPPADIPK.N	25.89	317	325
NKRF_HUMAN	E.IPPADIPK(+14.02).N	17.04	318	325
NKRF_HUMAN	I.PPADIPK.N	26.93	319	325
NKRF_HUMAN	I.PPADIPKNK.K	17.01	319	327
NKRF_HUMAN	K.KLLTDGYAC(+47.98)EVR.C	45.09	328	339
NKRF_HUMAN	K.LLTDGYAC(+47.98)EVR.C	58.7	329	339
NKRF_HUMAN	K.LLTDGYAC(-33.99)EVR.C	38.88	329	339
NKRF_HUMAN	K.LLTDGYAC(+70.04)EVR.C	27.42	329	339
NKRF_HUMAN	K.LLTDGYACEVR.C	25.35	329	339
NKRF_HUMAN	K.LLTDGYAC(+47.94)EVR.C	24.8	329	339
NKRF_HUMAN	K.LLT(+79.97)DGYACEVRCQNIYLTTGYAGS(+79.97)KNGSR.D	18.84	329	357
NKRF_HUMAN	K.LLTDGYAC(-20.03)EVR.C	15.33	329	339
NKRF_HUMAN	K.LLTDGY(+162.05)ACEVR(+14.02).C	15.29	329	339
NKRF_HUMAN	R.C(+25.00)QNIYLTTGYAGSK.N	66.49	340	353
NKRF_HUMAN	R.C(+47.98)QNIYLTTGYAGSK.N	63.29	340	353
NKRF_HUMAN	R.C(+70.04)QNIYLTTGYAGSK.N	40.77	340	353
NKRF_HUMAN	R.C(+87.03)QNIYLTTGYAGSK.N	38.85	340	353
NKRF_HUMAN	R.C(-33.99)QNIYLTTGYAGSK.N	19.86	340	353
NKRF_HUMAN	Y.LTTGYAGSK.N	45.94	345	353
NKRF_HUMAN	L.T(+79.97)TGYAGSK.N	20.36	346	353
NKRF_HUMAN	K.N(+43.01)GSRDRATELAVK.L	27.94	354	366
NKRF_HUMAN	K.NGSRD(+45.99)RATELAVK.L	17.65	354	366
NKRF_HUMAN	R.DRATELAVK.L	59.49	358	366
NKRF_HUMAN	R.DRATELAVK(-.98)(+14.02).L	19.12	358	366
NKRF_HUMAN	R.DRAT(+79.97)ELAVK(+27.99).L	17.58	358	366
NKRF_HUMAN	R.ATELAVK.L	58.54	360	366
NKRF_HUMAN	R.A(+42.01)TELAVK.L	43.33	360	366
NKRF_HUMAN	R.ATELAVK(+27.99)LLQK.R	42.42	360	370
NKRF_HUMAN	R.AT(-18.01)ELAVK.L	39.5	360	366
NKRF_HUMAN	R.ATELAVKLLQK.R	38.36	360	370

NKRF_HUMAN	R.ATELAVK(+14.02)LLQK(+14.02).R	24.46	360	370
NKRF_HUMAN	R.A(+42.01)T(-18.01)ELAVKLLQK.R	23.13	360	370
NKRF_HUMAN	A.T(-18.01)ELAVKLLQK.R	20.21	361	370
NKRF_HUMAN	A.LK(+14.96)PPEDLVVLGKDASGQPIFNASAK.H	25.44	405	429
NKRF_HUMAN	V.VLGKDAS(+79.97)GQPIFNASAK.H	17.77	413	429
NKRF_HUMAN	V.LGKD(+356.19)ASGQPIFNASAK.H	16.76	414	429
NKRF_HUMAN	K.DASGQPIFNASAK.H	94.52	417	429
NKRF_HUMAN	K.D(+42.01)ASGQPIFNASAK.H	66.8	417	429
NKRF_HUMAN	K.DASGQPIFN(+.98)ASAK.H	58.84	417	429
NKRF_HUMAN	K.DASGQ(+.98)PIFNASAK.H	57.94	417	429
NKRF_HUMAN	K.DAS(-18.01)GQPIFNASAK.H	38.01	417	429
NKRF_HUMAN	K.D(-18.01)ASGQPIFNASAK.H	24.97	417	429
NKRF_HUMAN	K.D(+27.99)ASGQ(+.98)PIFNASAK.H	19.19	417	429
NKRF_HUMAN	K.D(+21.98)ASGQPIFNASAK.H	15.1	417	429
NKRF_HUMAN	D.ASGQPIFNASAK.H	40.54	418	429
NKRF_HUMAN	N.DAIGILNNSASFNK.M	16.17	442	455
NKRF_HUMAN	D.AIGILNNS(+79.97)AS(+79.97)FNK.M	16.94	443	455
NKRF_HUMAN	N.SASFNKM(+15.99)SIEYK.Y	16.93	450	461
NKRF_HUMAN	A.SFNK(+27.99)MSIEYK.Y	19.3	452	461
NKRF_HUMAN	K.M(+15.99)SIEYKYEM(+15.99)M(+15.99)PNR.T	65.97	456	468
NKRF_HUMAN	K.M(+15.99)SIEYKYEMM(+31.99)PNR.T	49.47	456	468
NKRF_HUMAN	K.M(+15.99)SIEYKYEMMPNR(+31.99).T	21.59	456	468
NKRF_HUMAN	K.YEM(+15.99)M(+15.99)PNR.T	73.04	462	468
NKRF_HUMAN	K.YEM(+15.99)M(+31.99)PNR.T	49.44	462	468
NKRF_HUMAN	K.YEM(+15.99)MPNR.T	47.89	462	468
NKRF_HUMAN	K.Y(+79.97)EM(+15.99)MPNR.T	18.36	462	468
NKRF_HUMAN	K.Y(+79.97)EMMPNR(+14.02)T(+79.97)WRCR.V	16.8	462	473
NKRF_HUMAN	K.Y(+42.01)EMM(+15.99)PNRT(+79.97)WRCR.V	15.65	462	473
NKRF_HUMAN	K.Y(+79.97)EMM(+15.99)PNRTWRCR.V	15.53	462	473
NKRF_HUMAN	R.CRVFLQDHCLAEGYGT(+79.97)K.K	17.78	472	488
NKRF_HUMAN	R.VFLQDHC(+47.98)LAEGYGTK.K	71.37	474	488
NKRF_HUMAN	R.V(+42.01)F(+31.99)LQDHCLAEGYGTK.K	50.43	474	488
NKRF_HUMAN	R.VFLQDHC(+47.98)LAEGYGTKK.T	45.02	474	489
NKRF_HUMAN	R.V(+42.01)FLQDHC(+31.99)LAEGYGTK.K	42.97	474	488

NKRF_HUMAN	R.VFLQDHC(+47.94)LAEGYGTK.K	35.77	474	488
NKRF_HUMAN	R.VFLQDH(+79.97)CLAEGYGTK.K	29.31	474	488
NKRF_HUMAN	R.VFLQDHC(+58.01)LAEGYGTK.K	26.98	474	488
NKRF_HUMAN	R.VFLQDH(+77.91)CLAEGYGTK.K	17.02	474	488
NKRF_HUMAN	H.C(+25.00)LAEGYGTK.K	22.8	480	488
NKRF_HUMAN	C.LAEGYGTK.K	28.13	481	488
NKRF_HUMAN	K.TSKHAAADEALK.I	54.39	490	501
NKRF_HUMAN	K.T(+79.97)S(+79.97)KHAAADEALK.I	41.18	490	501
NKRF_HUMAN	K.TSK(+27.99)HAAADEALK.I	38.43	490	501
NKRF_HUMAN	K.T(+27.99)S(+79.97)KHAAADEALK.I	16.42	490	501
NKRF_HUMAN	K.T(+79.97)SKHAAADEALK.I	15.45	490	501
NKRF_HUMAN	K.HAAADEALK.I	80.43	493	501
NKRF_HUMAN	K.H(+42.01)AAADEALK.I	65.48	493	501
NKRF_HUMAN	K.H(+42.01)AAADEALKILQ(+.98)K.T	45.45	493	505
NKRF_HUMAN	K.H(+26.02)AAADEALK.I	42.25	493	501
NKRF_HUMAN	K.HAAADEALK(+31.99).I	32.57	493	501
NKRF_HUMAN	K.HAAADEALK(+14.96)I.L	29.07	493	502
NKRF_HUMAN	K.HAAADEALKILQK.T	29.07	493	505
NKRF_HUMAN	K.HAAADEALK(-.98).I	28.9	493	501
NKRF_HUMAN	K.H(+70.04)AAADEALK.I	24.48	493	501
NKRF_HUMAN	K.HAAAD(+31.97)EALK.I	24.4	493	501
NKRF_HUMAN	K.H(+42.01)AAADEALKILQK(-.98).T	23.81	493	505
NKRF_HUMAN	K.HAAADEALK(-2.02).I	23.12	493	501
NKRF_HUMAN	K.HAAADE(+129.04)AL.K	16.66	493	500
NKRF_HUMAN	K.HAAAD(+45.99)EALK.I	16.54	493	501
NKRF_HUMAN	K.H(+226.08)AAADEALK.I	15.86	493	501
NKRF_HUMAN	K.H(+42.01)AAADEALK(-.98).I	15.45	493	501
NKRF_HUMAN	K.HAAADEALK(+14.02).I	15.42	493	501
NKRF_HUMAN	H.A(+149.03)AADEALK.I	21.81	494	501
NKRF_HUMAN	D.EALKILQK.T	26.23	498	505
NKRF_HUMAN	K.ILQK(+27.99)TQPTYPSVK.S	55.33	502	514
NKRF_HUMAN	K.ILQKTQPTYPSVK.S	27.07	502	514
NKRF_HUMAN	K.ILQK(+31.99)TQPTYPSVK.S	21.48	502	514
NKRF_HUMAN	K.ILQKT(+79.97)QPTYPSVK.S	17.13	502	514

NKRF_HUMAN	K.ILQ(+.98)KT(+79.97)QPTYPSVK.S	16.18	502	514
NKRF_HUMAN	K.TQPTYPSVK.S	50.51	506	514
NKRF_HUMAN	K.T(+79.97)QPTYPS(+79.97)VK.S	22.28	506	514
NKRF_HUMAN	K.TQ(+.98)PTYPSVK.S	18.71	506	514
NKRF_HUMAN	K.T(+79.97)QPTYPSVK(+42.01).S	15.44	506	514
NKRF_HUMAN	K.T(-2.02)QPTYPSVK.S	15	506	514
NKRF_HUMAN	Q.PTYPSVK.S	22.16	508	514
NKRF_HUMAN	K.S(+79.97)SQCHT(+79.97)GSSPR.G	24.03	515	525
NKRF_HUMAN	K.S(+79.97)SQCHTGS(+79.97)SPR.G	17.79	515	525
NKRF_HUMAN	K.SS(+162.05)QCHT(+79.97)GSSPR.G	17.09	515	525
NKRF_HUMAN	K.S(+27.99)SQCHTGSSPR.G	16.59	515	525
NKRF_HUMAN	K.S(+79.97)S(+79.97)QCHTGSSPR.G	16.05	515	525
NKRF_HUMAN	K.DIKDLVVYENSSNPV(-.98).C	34.6	532	546
NKRF_HUMAN	K.DLVVYENSSNPVC(+47.94)TLNDTAQFNR.M	34.39	535	557
NKRF_HUMAN	K.DLVVYENSSNPVCT(+87.05)LNDTAQFNR.M	30.35	535	557
NKRF_HUMAN	K.DLVVYENSSNPVC(+47.98)TLNDTAQFNR.M	27.94	535	557
NKRF_HUMAN	K.DLVVYENSSNPV(-.98).C	27.81	535	546
NKRF_HUMAN	T.L(+127.06)NDTAQFNR.M	27.46	549	557
NKRF_HUMAN	A.Q(+42.01)FNRM(+15.99)TVEYVYER.M	26.73	554	566
NKRF_HUMAN	A.QFNRMTVEYVYER.M	22.25	554	566
NKRF_HUMAN	R.M(+15.99)TVEYVYER.M	85.14	558	566
NKRF_HUMAN	R.M(+31.99)TVEYVYER.M	58.49	558	566
NKRF_HUMAN	R.MTVEYVYER.M	56.29	558	566
NKRF_HUMAN	R.M(+42.01)(+15.99)TVEYVYER.M	50.74	558	566
NKRF_HUMAN	R.M(+42.01)TVEYVYER.M	50.43	558	566
NKRF_HUMAN	R.M(+15.99)TVEYVY(+31.99)ER.M	38.74	558	566
NKRF_HUMAN	R.M(+15.99)TVEY(+31.99)VYER.M	37.66	558	566
NKRF_HUMAN	R.M(+15.99)T(-18.01)VEYVYER.M	28	558	566
NKRF_HUMAN	R.M(+27.99)TVEYVYER.M	24.01	558	566
NKRF_HUMAN	R.MT(+14.02)VEYVYER.M	22.34	558	566
NKRF_HUMAN	R.M(+15.99)TVEYVYER(+28.03).M	18.99	558	566
NKRF_HUMAN	R.MT(+13.03)VEYVYER.M	18.79	558	566
NKRF_HUMAN	R.MTVE(+14.02)YVYER.M	16.48	558	566
NKRF_HUMAN	R.MTGLRWKCK.V	19.27	567	575

NKRF_HUMAN	K.VILESEVIAEAVGVK.K	105.98	576	590
NKRF_HUMAN	K.VILESEVIAEAVGVKK.T	94.79	576	591
NKRF_HUMAN	K.VILES(-2.02)EVIAEAVGVKK.T	18.7	576	591
NKRF_HUMAN	V.I(+87.03)LESEVIAEAVGVKK.T	20.51	577	591
NKRF_HUMAN	V.ILES(+95.94)EVIAEAVGVKK.T	17.86	577	591
NKRF_HUMAN	I.LES(+238.23)EVIAEAVGVKK.T	15.15	578	591
NKRF_HUMAN	K.TVKYEAAGEAVK.T	87.63	592	603
NKRF_HUMAN	K.TVK(+27.99)YEAAGEAVK.T	53.09	592	603
NKRF_HUMAN	K.TVKYE(+21.98)AAGEAVK.T	32.65	592	603
NKRF_HUMAN	K.T(-18.01)VK(+42.01)YEAAGEAVK.T	20.88	592	603
NKRF_HUMAN	T.VK(+14.02)Y(+79.97)EAAGEAVK.T	20.34	593	603
NKRF_HUMAN	V.KYEAAGEAVK.T	43.84	594	603
NKRF_HUMAN	K.YEAAGEAVK.T	70.4	595	603
NKRF_HUMAN	K.Y(+15.99)EAAGEAVK.T	43.2	595	603
NKRF_HUMAN	K.Y(+42.01)EAAGEAVK.T	41.74	595	603
NKRF_HUMAN	K.YE(+21.98)AAGEAVK.T	39.41	595	603
NKRF_HUMAN	K.YEAAGE(+21.98)AVK.T	32.02	595	603
NKRF_HUMAN	K.Y(-2.02)EAAGEAVK.T	22.56	595	603
NKRF_HUMAN	K.YEAAGEAVK(+28.03).T	22.29	595	603
NKRF_HUMAN	K.Y(-94.04)EAAGEAVK.T	20.47	595	603
NKRF_HUMAN	K.Y(+15.01)EAAGEAVK.T	20.19	595	603
NKRF_HUMAN	K.Y(+42.01)(-18.01)EAAGEAVK.T	19.28	595	603
NKRF_HUMAN	K.Y(+79.97)EAAGEAVK(+21.98).T	15.53	595	603
NKRF_HUMAN	K.TLKKT(+79.97)Q(+.98)PTVIN(+.98)NLK.K	23.7	604	617
NKRF_HUMAN	K.T(+79.97)LKKTQ(+.98)PTVIN(+.98)NLK.K	16.28	604	617
NKRF_HUMAN	K.KTQPTVINNLKK.G	80.04	607	618
NKRF_HUMAN	K.KTQPTVINNLK.K	63.62	607	617
NKRF_HUMAN	K.KTQPTVIN(+.98)NLK.K	49.08	607	617
NKRF_HUMAN	K.KTQPTVIN(+.98)NLKK.G	47.54	607	618
NKRF_HUMAN	K.KTQ(+.98)PTVINNLK(+43.99).K	27.14	607	617
NKRF_HUMAN	K.KTQ(+.98)PTVINNLKK.G	21.14	607	618
NKRF_HUMAN	K.K(+42.01)(+31.99)TQPTVINNLKK.G	20.55	607	618
NKRF_HUMAN	K.KTQPT(-18.01)VINNLKK.G	19.14	607	618
NKRF_HUMAN	K.KTQPT(+79.97)VINNLK.K	17.75	607	617

NKRF_HUMAN	K.KTQPTVINN(+.98)LKK.G	15.56	607	618
NKRF_HUMAN	K.TQPTVINNLK.K	61.43	608	617
NKRF_HUMAN	K.TQPTVINNLKK.G	59.41	608	618
NKRF_HUMAN	K.TQPTVIN(+.98)NLKK.G	57.91	608	618
NKRF_HUMAN	K.TQPTVINN(+.98)LK.K	48.94	608	617
NKRF_HUMAN	K.TQPTVIN(+.98)NLK.K	48.71	608	617
NKRF_HUMAN	K.T(+42.01)QPTVINNLK.K	37.55	608	617
NKRF_HUMAN	K.TQ(+.98)PTVINNLKK.G	34.35	608	618
NKRF_HUMAN	K.TQ(+.98)PTVINNLK.K	34.17	608	617
NKRF_HUMAN	K.TQ(+.98)PTVIN(+.98)NLK.K	27.53	608	617
NKRF_HUMAN	K.TQ(+.98)PTVINNLK(-.98).K	21.2	608	617
NKRF_HUMAN	K.T(+42.01)(-18.01)QPTVINNLK.K	19.11	608	617
NKRF_HUMAN	K.TQPTVINNLK(+114.04)K.G	18.39	608	618
NKRF_HUMAN	K.T(+79.97)QPT(+79.97)VINNLKK.G	16.52	608	618
NKRF_HUMAN	K.T(+79.97)QPT(+79.97)VINNLK.K	16.16	608	617
NKRF_HUMAN	K.T(+79.97)QPT(+79.97)VINN.L	15.79	608	615
NKRF_HUMAN	Q.PTVINNLKK.G	56.15	610	618
NKRF_HUMAN	T.VINNLKK.G	21.34	612	618
NKRF_HUMAN	I.NNLK(+31.99)KGAVEDVISR.N	15.12	614	627
NKRF_HUMAN	K.KGAVEDVISR.N	84.91	618	627
NKRF_HUMAN	K.K(+26.02)GAVEDVISR.N	48.16	618	627
NKRF_HUMAN	K.K(+28.03)GAVEDVISR.N	34.05	618	627
NKRF_HUMAN	K.KGAVEDVIS(+79.96)R.N	18.51	618	627
NKRF_HUMAN	K.K(+42.01)(+42.01)GAVEDVISR.N	17.43	618	627
NKRF_HUMAN	K.K(+42.01)GAVEDVISR.N	17.33	618	627
NKRF_HUMAN	K.GAVEDVISR.N	64.05	619	627
NKRF_HUMAN	K.G(+42.01)AVEDVISR.N	36.37	619	627
NKRF_HUMAN	K.GAVEDVISR(+.98).N	28.88	619	627
NKRF_HUMAN	K.GAVE(+21.98)DVISR.N	28.82	619	627
NKRF_HUMAN	K.G(+42.01)AVED(-18.01)VISR.N	28.37	619	627
NKRF_HUMAN	K.GAVE(+28.03)DVISR.N	24.85	619	627
NKRF_HUMAN	K.GAVEDVISRN(+.98)EIQGR.S	19.01	619	633
NKRF_HUMAN	K.GAVEDVIS(+79.97)R.N	17.84	619	627
NKRF_HUMAN	A.VEDVISR.N	37.21	621	627

NKRF_HUMAN	E.I(+27.99)QGRSAEEAYK.Q	16.98	630	640
NKRF_HUMAN	I.QGR(+54.01)SAEEAYK.Q	15.85	631	640
NKRF_HUMAN	R.SAEEAYKQQIK.E	88.22	634	644
NKRF_HUMAN	R.SAEEAYKQQIKEDNIGNQLLR.K	78.72	634	654
NKRF_HUMAN	R.SAEEAYK.Q	62.88	634	640
NKRF_HUMAN	R.S(-2.02)AEEAYKQQIK.E	26.09	634	644
NKRF_HUMAN	R.SAEEAYKQ(+.98)QIK.E	25.24	634	644
NKRF_HUMAN	R.SAEEAYKQQ(+.98)IK.E	21.67	634	644
NKRF_HUMAN	R.SAEE(+28.03)AYK.Q	20.03	634	640
NKRF_HUMAN	R.SAEEAYKQ(+.98)QIKEDNIGNQLLR.K	19.19	634	654
NKRF_HUMAN	R.SAEEAYKQQIKEDN(+.98)IGNQLLR.K	18.55	634	654
NKRF_HUMAN	R.SAEEAYKQ(+.98)Q(+.98)IK.E	17.84	634	644
NKRF_HUMAN	R.SAEEAYK(+28.03).Q	16.57	634	640
NKRF_HUMAN	R.SAEEAYK(+14.02).Q	16.22	634	640
NKRF_HUMAN	S.AEEAYKQQIK.E	48.95	635	644
NKRF_HUMAN	A.EEAYKQQIK.E	55.34	636	644
NKRF_HUMAN	Y.KQQIKE(+21.98)DNIGNQLLR.K	28.07	640	654
NKRF_HUMAN	K.QQIKEDNIGNQLLR.K	96.32	641	654
NKRF_HUMAN	K.Q(-17.03)QIKEDNIGNQLLR.K	69.33	641	654
NKRF_HUMAN	K.QQIKEDNIGNQLLRK.M	49.83	641	655
NKRF_HUMAN	K.Q(-17.03)QIK(+42.01)EDNIGNQLLR.K	44.71	641	654
NKRF_HUMAN	K.QQIKED(-18.01)NIGNQLLR.K	40.93	641	654
NKRF_HUMAN	K.QQIK(+28.03)EDNIGNQLLR.K	29.45	641	654
NKRF_HUMAN	K.QQIKEDNIGN(+.98)QLLR.K	28.33	641	654
NKRF_HUMAN	K.QQIK(+26.02)EDNIGNQLLR.K	27.31	641	654
NKRF_HUMAN	K.Q(+42.01)Q(+.98)IKEDNIGNQLLR.K	27.04	641	654
NKRF_HUMAN	K.Q(+42.01)QIKEDN(+.98)IGNQLLR.K	25.68	641	654
NKRF_HUMAN	K.QQ(+.98)IKEDNIGNQLLR.K	25.11	641	654
NKRF_HUMAN	K.Q(+42.01)QIKEDNIGNQLLR.K	24.23	641	654
NKRF_HUMAN	K.Q(-17.03)QIKEDN(+.98)IGNQLLR.K	22.18	641	654
NKRF_HUMAN	K.QQIKED(+79.97)NIGNQLLR.K	19.34	641	654
NKRF_HUMAN	K.QQIK(+27.99)EDNIGNQLLR.K	19.25	641	654
NKRF_HUMAN	K.QQIK(+42.01)EDNIGNQLLR.K	19.13	641	654
NKRF_HUMAN	K.QQIKEDN(+.98)IGN(+.98)QLLR.K	18.96	641	654

NKRF_HUMAN	K.Q(+.98)Q(+.98)IKEDN(+.98)IGNQLLR.K	17.29	641	654
NKRF_HUMAN	K.EDNIGNQLLR.K	85.14	645	654
NKRF_HUMAN	K.E(+42.01)DNIGNQLLR.K	48.03	645	654
NKRF_HUMAN	K.ED(-18.01)NIGNQLLR.K	47.88	645	654
NKRF_HUMAN	K.EDNIGNQ(+.98)LLR.K	47.36	645	654
NKRF_HUMAN	K.E(+42.01)D(-18.01)NIGNQLLR.K	46.87	645	654
NKRF_HUMAN	K.E(-18.01)DNIGNQLLR.K	39.63	645	654
NKRF_HUMAN	K.E(+42.01)DN(+.98)IGNQLLR.K	38.17	645	654
NKRF_HUMAN	K.EDN(+.98)IGNQLLR.K	33.34	645	654
NKRF_HUMAN	K.E(+43.01)DN(+.98)IGNQLLRK.M	16.84	645	655
NKRF_HUMAN	D.NIGNQLLR.K	17.05	647	654
NKRF_HUMAN	R.K(+31.99)MGWTGGGLGK.S	35.91	655	665
NKRF_HUMAN	R.KMGW(+19.99)TGGGLGK.S	25.98	655	665
NKRF_HUMAN	K.M(+15.99)GW(+31.99)TGGGLGK.S	53.74	656	665
NKRF_HUMAN	K.M(+15.99)GW(+15.99)TGGGLGK.S	48.11	656	665
NKRF_HUMAN	K.MGW(+19.99)TGGGLGK.S	39.62	656	665
NKRF_HUMAN	K.M(+15.99)GW(+43.99)TGGGLGK.S	39.07	656	665
NKRF_HUMAN	K.M(+31.99)GWTGGGLGK.S	18.67	656	665
NKRF_HUMAN	K.M(+27.99)GWTGGGLGK.S	17.26	656	665
NKRF_HUMAN	K.SGEGIREPISVK.E	70.57	666	677
NKRF_HUMAN	K.SGEGIREPISVKEQHK.R	32.98	666	681
NKRF_HUMAN	K.SGEGIREPISVK(+109.05)EQHK.R	28.85	666	681
NKRF_HUMAN	K.S(+42.01)GEGIREPISVK.E	23.7	666	677
NKRF_HUMAN	K.S(+95.94)GEGIREPISVK.E	21.44	666	677
NKRF_HUMAN	K.S(+42.01)GEGIR(+31.99)EPISVK.E	20.43	666	677
NKRF_HUMAN	S.GEGIREPISVK.E	43.38	667	677
NKRF_HUMAN	G.EGIREPISVK.E	36.6	668	677
NKRF_HUMAN	E.GIREPISVK.E	37.99	669	677
NKRF_HUMAN	R.EPIS(+79.97)VKE.Q	18.54	672	678
NKRF_HUMAN	R.EPISVKEQHK(+226.08)R.E	16.47	672	682
NKRF_HUMAN	K.EQHKR(+14.02)EGLGLDVER.V	31.12	678	691
NKRF_HUMAN	K.R(+14.02)E(+43.99)GLGLDVER.V	17.43	682	691
NKRF_HUMAN	R.EGLGLDVER.V	72.37	683	691
NKRF_HUMAN	R.E(-18.01)GLGLDVER.V	62.77	683	691

NKRF_HUMAN	R.E(+42.01)GLGLDVER.V	42.22	683	691
NKRF_HUMAN	R.E(+53.92)GLGLDVER.V	34.62	683	691
NKRF_HUMAN	R.EGL(+53.97)GLDVER.V	34.41	683	691
NKRF_HUMAN	R.EGLGLDVE(+28.03)R.V	31.96	683	691
NKRF_HUMAN	R.E(+43.01)GLGLDVER.V	26.59	683	691
NKRF_HUMAN	R.E(+21.98)GLGLDVER.V	18.33	683	691
NKRF_HUMAN	R.E(+37.96)GLGLDVER.V	17.09	683	691
NKRF_HUMAN	R.EGLGLDVER(+28.03).V	15.83	683	691
NKRF_HUMAN	G.LGLDVER.V	23.03	685	691
NKRF_HUMAN	V.ERVNKIAK.R	26.28	690	697
NKRF_HUMAN	R.VNK(+42.01)IAK(+31.99)RDIEQIIR.N	16.21	692	705
NKRF_HUMAN	K.RDIEQIIR.N	57.71	698	705
NKRF_HUMAN	K.R(+42.01)DIEQIIR.N	36.26	698	705
NKRF_HUMAN	K.R(+14.02)DIEQIIR.N	17.31	698	705
NKRF_HUMAN	K.RDIEQ(+.98)IIR.N	15.2	698	705
NKRF_HUMAN	K.R(+42.01)DIEQ(+.98)IIR.N	15.1	698	705
NKRF_HUMAN	R.DIEQIIR.N	60.77	699	705
NKRF_HUMAN	R.D(+42.01)IEQIIR.N	38.51	699	705
NKRF_HUMAN	R.DIEQ(+.98)IIR.N	27.66	699	705
NKRF_HUMAN	R.D(+27.99)IEQIIR.N	20.68	699	705
NKRF_HUMAN	R.D(-18.01)IEQIIR.N	18.51	699	705
NKRF_HUMAN	D.IEQIIRNYAR.S	16.29	700	709
NKRF_HUMAN	R.NYARS(+79.97)ES(+79.97)HTDLTFSR.E	38.73	706	720
NKRF_HUMAN	R.NYARSES(+79.97)HT(+79.97)DLTFSR.E	19.42	706	720
NKRF_HUMAN	R.NYARSESHT(+79.97)DLTFSR.E	16.17	706	720
NKRF_HUMAN	R.SESHTDLTFSR.E	101.1	710	720
NKRF_HUMAN	R.SESH(+14.02)TDLTFSR.E	54.21	710	720
NKRF_HUMAN	R.S(+42.01)ESHTDLTFSR.E	51.42	710	720
NKRF_HUMAN	R.SESHT(+14.02)DLTFSR.E	38.3	710	720
NKRF_HUMAN	R.SESHTD(-18.01)LTFSR.E	27.66	710	720
NKRF_HUMAN	R.SESHTDLTF.S	27.1	710	718
NKRF_HUMAN	R.SES(+14.02)HTDLTFSR.E	26.45	710	720
NKRF_HUMAN	R.SESHT(+79.97)DLTFSR.E	25.3	710	720
NKRF_HUMAN	R.SE(+43.99)SHTDLTFSR.E	25.04	710	720

NKRF_HUMAN	R.S(-2.02)ESHTDLTFSR.E	24.65	710	720
NKRF_HUMAN	R.S(+79.97)ESHT(+79.97)DLTFSR.E	23.19	710	720
NKRF_HUMAN	R.SESHTD(+31.97)LTFSR.E	22.88	710	720
NKRF_HUMAN	R.SESHTDLT(+79.97)FS(+79.97)R.E	22.63	710	720
NKRF_HUMAN	R.SESHT(+42.01)DLTFSR.E	22.52	710	720
NKRF_HUMAN	R.SESHTD(+45.99)LTFSR.E	22.29	710	720
NKRF_HUMAN	R.SESHT(-18.01)DLTFSR.E	20.71	710	720
NKRF_HUMAN	R.S(+42.01)ES(-18.01)HTDLTFSR.E	19.79	710	720
NKRF_HUMAN	R.SESHTDLTF(+31.99)SR.E	17.76	710	720
NKRF_HUMAN	R.SESHT(+79.97)DLT(+79.97)FSR.E	17.68	710	720
NKRF_HUMAN	R.S(+79.97)ESHTDLT(+79.97)FSR.E	17.53	710	720
NKRF_HUMAN	R.S(+43.01)ESHTDLTFSR.E	17.26	710	720
NKRF_HUMAN	R.SESHTD(+43.99)LTFSR.E	16.71	710	720
NKRF_HUMAN	R.S(+79.97)ESHTDLTFSR.E	16.5	710	720
NKRF_HUMAN	R.S(-18.01)ESHTDLTFSR.E	15.67	710	720
NKRF_HUMAN	R.SESHTD(+6.01)LTFSR.E	15.25	710	720
NKRF_HUMAN	S.ESHTDLTFSR.E	60.79	711	720
NKRF_HUMAN	E.SHTDLTFSR.E	52.48	712	720
NKRF_HUMAN	E.S(-2.02)HTDLTFSR.E	18.3	712	720
NKRF_HUMAN	S.H(+42.01)TDLTFSR.E	16.21	713	720
NKRF_HUMAN	L.TFSRELTNDERK.Q	15.75	717	728
NKRF_HUMAN	R.ELTNDERK.Q	62.8	721	728
NKRF_HUMAN	R.ELTNDER.K	59.88	721	727
NKRF_HUMAN	R.E(-18.01)LTNDERK.Q	42.13	721	728
NKRF_HUMAN	R.ELT(-18.01)NDER.K	30.08	721	727
NKRF_HUMAN	R.E(+43.01)LTNDERK.Q	27.31	721	728
NKRF_HUMAN	R.E(+42.01)LTNDERK.Q	24.71	721	728
NKRF_HUMAN	R.ELT(-18.01)NDERK.Q	21.2	721	728
NKRF_HUMAN	R.ELT(+79.97)NDER.K	21.08	721	727
NKRF_HUMAN	R.E(-18.01)LTNDER.K	17.65	721	727
NKRF_HUMAN	R.ELTN(+.98)DERK.Q	15.62	721	728
NKRF_HUMAN	R.KQIHQIAQK.Y	80.27	728	736
NKRF_HUMAN	R.K(+42.01)QIHQIAQK.Y	35.83	728	736
NKRF_HUMAN	R.KQ(+.98)IHQIAQK.Y	21.65	728	736

NKRF_HUMAN	R.K(+14.02)QIHQ(+.98)IAQK.Y	16.23	728	736
NKRF_HUMAN	R.KQIHQIAQK(+31.99).Y	15.88	728	736
NKRF_HUMAN	K.QIHQIAQK.Y	62.2	729	736
NKRF_HUMAN	K.QIHQIAQ(+.98)K.Y	47.96	729	736
NKRF_HUMAN	K.Q(-17.03)IHQIAQK.Y	35.03	729	736
NKRF_HUMAN	K.Q(-17.03)IHQIAQ(+.98)K.Y	27.67	729	736
NKRF_HUMAN	K.QIH(+14.02)QIAQK.Y	26.14	729	736
NKRF_HUMAN	Q.IHQIAQK.Y	50.17	730	736
NKRF_HUMAN	K.YGLKSKS(+79.97)H(+15.99)GVGHDR.Y	15.7	737	750
NKRF_HUMAN	L.K(+42.01)SK(+42.01)SHGVGHDR.Y	15.37	740	750
NKRF_HUMAN	K.SKSH(+14.02)GVGHDR.Y	42.73	741	750
NKRF_HUMAN	K.SKSHGVGHDR.Y	39.83	741	750
NKRF_HUMAN	K.S(+42.01)K(+43.99)SHGVGHDR.Y	17.84	741	750
NKRF_HUMAN	K.SK(+31.99)SHGVGHDR.Y	16.78	741	750
NKRF_HUMAN	K.SHGVGHDR.Y	81.59	743	750
NKRF_HUMAN	K.S(+42.01)HGVGHDR.Y	30.93	743	750
NKRF_HUMAN	K.SHGVGHDR(+31.99).Y	25.17	743	750
NKRF_HUMAN	K.S(+79.97)H(+15.99)GVGHDR.Y	25.15	743	750
NKRF_HUMAN	K.SH(+42.01)GVGHDR.Y	17.13	743	750
NKRF_HUMAN	S.HGVGHDR.Y	56.31	744	750
NKRF_HUMAN	R.RKEDLLDQLK.Q	56.74	759	768
NKRF_HUMAN	R.RKEDLLDQL.K	18.49	759	767
NKRF_HUMAN	R.RK(+383.23)EDLLDQLK.Q	15.17	759	768
NKRF_HUMAN	R.KEDLLDQLK.Q	63.66	760	768
NKRF_HUMAN	R.K(+42.01)EDLLDQLK.Q	39.34	760	768
NKRF_HUMAN	R.KEDLLDQLKQ(+28.03).E	18.54	760	769
NKRF_HUMAN	R.KEDLLDQLK(+156.12).Q	17.1	760	768
NKRF_HUMAN	R.K(-1.03)EDLLDQLK.Q	16.54	760	768
NKRF_HUMAN	K.EDLLDQLK.Q	52.42	761	768
NKRF_HUMAN	K.ED(-18.01)LLDQLK.Q	23.2	761	768
NKRF_HUMAN	K.E(-18.01)DLLDQLK.Q	16.58	761	768
NKRF_HUMAN	K.QEGQVGHYELVM(+15.99)PQAN	76.57	769	784
NKRF_HUMAN	K.Q(-17.03)EGQVGHYELVM(+15.99)PQAN	61.04	769	784
NKRF_HUMAN	K.QEGQVGHYELVM(+31.99)PQAN	58.06	769	784

NKRF_HUMAN	K.QEGQVGHYELVM(+15.99)PQ(+.98)AN	38.37	769	784
NKRF_HUMAN	K.QEGQVGHYELVM(+15.99)PQAN(+.98)	37.71	769	784
NKRF_HUMAN	K.QEGQVGHYELVM(+15.99)PQ.A	36.72	769	782

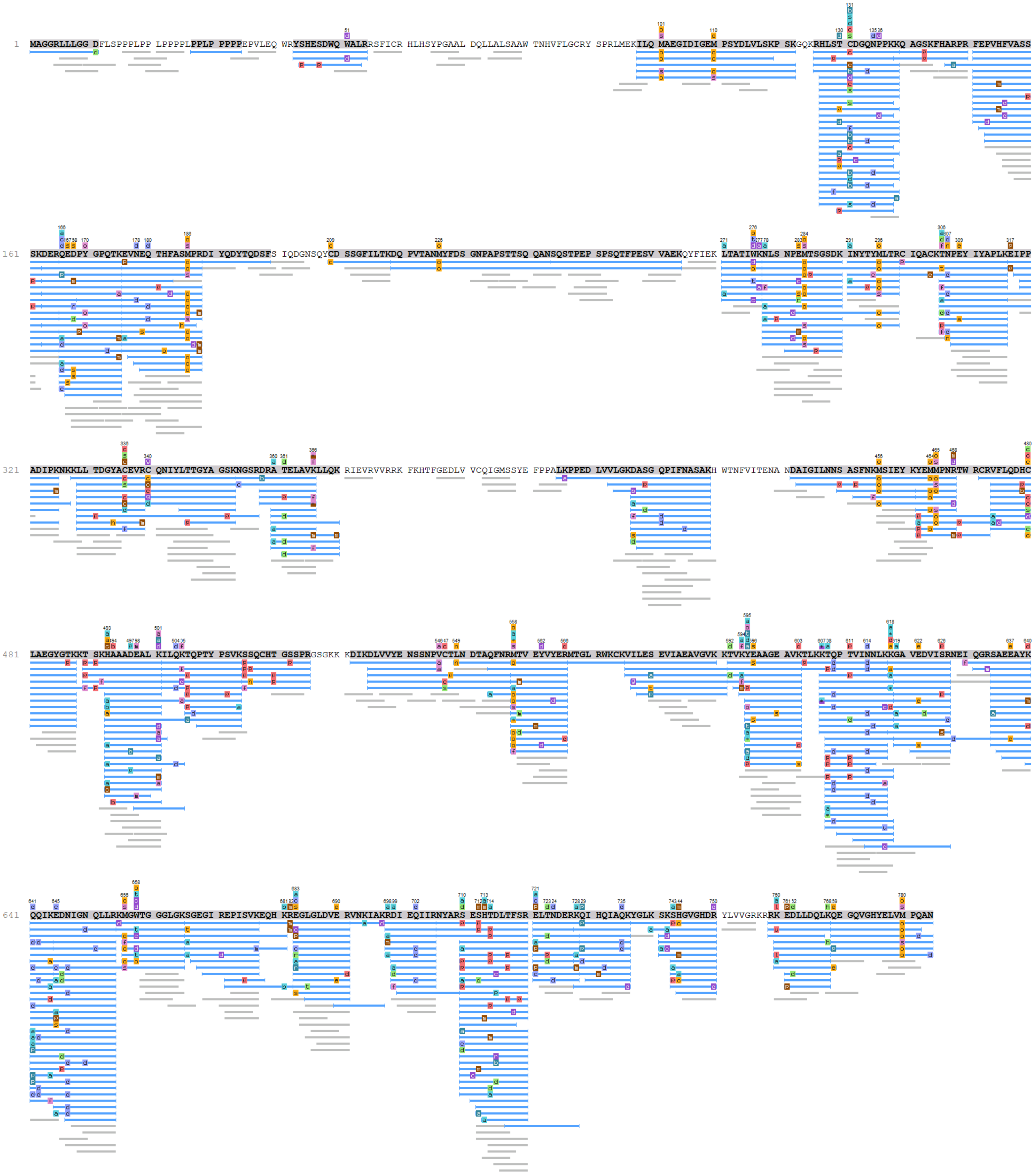
1 MAGGRLLLGG DFLSPPPLPP LPPPPLPPLP PPPPEPVLEQ WRYSHESDWQ WALRRSFICR HLHSYPGAAL DQLLALSAAW TNHVFLGCRY SPRLMEKILQ MAEGIDIGEM PSYDLVLSKP SKGQKRHLST CDGQNPPKKQ AGSKFHARPR FEPVHFVASS
161 SKDERQEDPY GPQTKEVNEQ THFASMPRDI YQDYTQDSFS IQDGNSQYCD SSGFILTKDQ PVTANMYFDS GNPAPSTTSQ QANSQSTPEP SPSQTFPESV VAEKQYFIEK LTATIWKNLS NPEMTSGSDK INYTYMLTRC IQACKTNPEY IYAPLKEIPP
321 ADIPKNKKLL TDGYACEVRC QNIYLTTGYA GSKNGSRDRA TELAVKLLQK RIEVRVVRRK FKHTFGEDLV VCQIGMSSYE FPPALKPPED LVVLGKDASG QPIFNASAKH WTNFVITENA NDAIGILNNS ASFNKMSIEY KYEMMPNRTW RCRVFLQDHC
481 LAEGYGTKKT SKHAAADEAL KILQKTQPTY PSVKSSQCHT GSSPRGSGKK KDIKDLVVYE NSSNPVCTLN DTAQFNRMTV EYVYERMTGL RWKCKVILES EVIAEAVGVK KTVKYEAAGE AVKTLKKTQP TVINNLKKGA VEDVISRNEI QGRSAEEAYK
641 QQIKEDNIGN QLLRKMGWTG GGLGKSGEGI REPISVKEQH KREGLGLDVE RVNKIAKRDI EQIIRNYARS ESHTDLTFSR ELTNDERKQI HQIAQKYGLK SKSHGVGHDR YLVVGRKRRK EDLLDQLKQE GQVGHYELVM PQAN
